# Golgi-apparatus genes related signature for predicting the progression-free interval of patients with papillary thyroid carcinoma

**DOI:** 10.1186/s12920-023-01485-z

**Published:** 2023-03-27

**Authors:** Rui Liu, Zhen Cao, Mengwei Wu, Xiaobin Li, Peizhi Fan, Ziwen Liu

**Affiliations:** 1 Department of Breast and Thyroid Surgery, Hunan Provincial People’s Hospital/The First Affiliated Hospital of Hunan Normal University, Changsha, 410005 China; 2 Department of General Surgery, Peking Union Medical College Hospital, Chinese Academy of Medical Sciences and Peking Union Medical College, Beijing, 100730 China

**Keywords:** Papillary thyroid carcinoma, Predictive model, The Cancer Genome Atlas Program, Golgi apparatus related genes, Nomogram

## Abstract

**Background:**

We aimed to build a novel model with golgi apparatus related genes (GaGs) signature and relevant clinical parameters for predicting progression-free interval (PFI) after surgery for papillary thyroid carcinoma (PTC).

**Methods:**

We performed a bioinformatic analysis of integrated PTC datasets with the GaGs to identify differentially expressed GaGs (DE-GaGs). Then we generated PFI-related DE-GaGs and established a novel GaGs based signature. After that, we validated the signature on multiple external datasets and PTC cell lines. Further, we conducted uni- and multivariate analyses to identify independent prognostic characters. Finally, we established a signature and clinical parameters-based nomogram for predicting the PFI of PTC.

**Results:**

We identified 260 DE-GaGs related to PFI in PTC. The functional enrichment analysis showed that the DE-MTGs were associated with an essential oncogenic glycoprotein biosynthetic process. Consequently, we established and optimized a novel 11 gene signature that could distinguish patients with poorer prognoses and predicted PFI accurately. The novel signature had a C-index of 0.78, and the relevant nomogram had a C-index of 0.79. Also, it was closely related to the pivotal clinical characters of and anaplastic potential in datasets and PTC cell lines. And the signature was confirmed a significant independent prognostic factor in PTC. Finally, we built a nomogram by including the signature and relevant clinical factors. Validation analysis showed that the nomogram’s efficacy was satisfying in predicting PTC’s PFI.

**Conclusion:**

The GaGs signature and nomogram were closely associated with PTC prognosis and may help clinicians improve the individualized prediction of PFI, especially for high-risk patients after surgery.

**Supplementary Information:**

The online version contains supplementary material available at 10.1186/s12920-023-01485-z.

## Background

Thyroid cancer (TC) has become the most commonly diagnosed endocrine tumor over the past decades [1]. Should the recent trends of TC prevail, it may become the fourth most common cancer in the United States by 2030 [2]. The most common and least aggressive histologic type of TC is papillary TC (PTC), comprising 80% of all cases. PTC is characterized by a favorable outcome after adequate total thyroidectomy, with or without regional lymphadenectomy [3]. However, one of the primary concerns after the initial surgery is a recurrent disease, which is 5.7% at five years and 9.4% at ten years, as Karl et al. reported in 52,173 PTC surgery patients [4]. Re-operations for the recurrent disease could result in a higher risk of surgical complications [5]. Clinical predictive models such as the American Thyroid Association (ATA) risk stratification have been widely used [6]. However, the clinical and pathological character-based models developed thus far do not reflect individual characteristics at the molecular level [7]. Therefore, novel prognostic tools for guiding personalized surveillance, especially for patients with a high risk of recurrence, are urgently needed. Developing a predictive model based on sensitive biomarkers would facilitate personalized monitoring, reducing the possibility of advanced, recurrent diseases in the postoperative follow-up period. Recently, progression in high-throughput sequencing has led to optimistic expectations about personalized medicine. Signatures based on biomarkers such as mRNA or lncRNA have great potential to predict cancer prognosis [8, 9]. These omics-based models can also reliably predict the prognosis of PTC [10, 11].

The Golgi apparatus is a processing and sorting hub in transporting and targeting soluble cargo proteins and lipids to different destinations in the cell [12]. Involved in fundamental molecular and cell biological processes that occur in cancer, such as cancer cell invasion, cell matrix adhesion, cancer angiogenesis, immune modulation and metastasis, accumulating reports and evidences indicated that the Golgi apparatus functioning disorders played pivotal roles in multiple human cancers including prostate cancer, breast cancer, gastric cancer and thyroid cancer [13–16]. Hence, abnormally functioned Golgi apparatus genes (GaGs) based on predictive models may be closely related to the prognosis of PTC. Therefore, we identified differentially expressed GaGs (DE-GaGs) after the intersection with the experimentally supported GaGs derived from MsigDB database [17]. Then we proposed a novel golgi apparatus related to the 11-gene signature and constructed a nomogram with relevant clinical factors. Validation analyses indicated the predicting ability of GaG signature and relevant prognostic model was satisfactory.

## Methods

### Obtain of TCGA-THCA RNA sequencing data and clinical information

We used Genomic Data Commons Application Programming Interface (GDC API) to download RNA sequencing data from The Cancer Genome Atlas Thyroid carcinoma (TCGA-THCA) up to 21 Jul 2019, including 507 PTC cases and relevant follow-up information. Transcript per million (TPM) transformation followed by base-2 logarithm normalization was applied. Cases with a follow-up period of less than a month were excluded. Considering the very low cancer-related death rate, we extracted progression-free interval (PFI) data from the University of California Santa Cruz (UCSC) Xena database as a specific survival outcome [10]. Both structural evidence (includes distant metastasis, locoregional recurrence, and new primary tumor) and biochemical evidence of recurrence was defined as progression. We also retrieved clinical and mutational data from the Cbioportal.

### Identification of DE-GaGs and GEO datasets acquisition

A differential gene expression analysis was applied based on all the 502 PTC cases with 58 normal thyroid tissues from TCGA-THCA dataset using the R package “EdgeR”. We identified DEGs according to the criteria of false discovery rate (FDR) < 0.05 and |Log2FC| >1 [18]. GaGs were extracted from Gene Set: “GOCC_GOLGI_APPARATUS” of the MsigDB database which curated 1613 golgi apparatus-related genes. After the intersection with the reliable DEGs, DE-GaGs were generated. After that, we searched the GEO database to obtain datasets including poorly or undifferentiated PTC. The keywords for the search included “Thyroid cancer,” “Homo sapiens,” “undifferentiated,” “poorly differentiated” and “anaplastic” The research focused on “cell lines,” and “xenografts” was excluded. Cases of childhood PTC, PTC in young adults, and radiation-induced PTC were also excluded. Raw data were normalized using the RMAExpress software [19]. Probe names were transformed into official symbols based on Thermo Fisher Scientific Inc’s annotation file. The median value was replaced if more than one probes to a single gene symbol.

### Functional enrichment analysis

We carried out functional enrichment analyses using the “clusterProfiler” package of R to explore the potential enriched function of the DE-GaGs [20, 21]. The Benjamini and Hochberg method was used for FDR correction, defining adjusted p < 0.05 as statistically significant.

### Construction and verification of the novel GaGs based signature

According to the general assumption in deep learning that more training data leads to better performance, we randomly divided the TCGA-THCA dataset into training and testing datasets in the ratio of 0.8 [22]. We used the univariate Cox regression model to identify the DE-GaGs that were significantly associated (p < 0.05) with PFI in the training set. The PFI-related DE-GaGs were further included. Then we applied the Least absolute shrinkage and selection operator (LASSO) analysis, often used in high-dimensional data to reduce the dimension by penalizing the number of regression coefficients, to further select valid variables using the “glmnet” R package [23]. The “cv.glmnet” function of the package is used to build the model. Cross-validation used different lambda values to observe the model error. Then cv plot was generated, and the best lambda value was chosen. Then a panel of gene signature was found. The predictive efficacy of the signature was then assessed with the ROC curve and C-index by the “timeROC” package and the “survcomp” package of the R software [24].

### External validation of 11-gene signature in GEO datasets

The expression pattern of GaG-based signature from three datasets (GSE29265, GSE33630 and GSE76039 [25]) including PTC, anaplastic thyroid carcinoma (ATC) and poorly differentiated thyroid carcinoma (PDTC) samples were extracted. Each sample’s risk score was generated to evaluate the potential clinical utility of the 11-gene signature. P-value of < 0.05 as statistically significant. All the GSE datasets were obtained in Gene Expression Omnibus (GEO).

### Cell culture and lysis

Normal human thyroid follicular cell line Nthy-ori 3.1 [26]and PTC cell line KTC-1 [27] were kindly provided by Dr. Lv from Hunan Key Laboratory of Organ Fibrosis. Nthy-ori 3.1 and KTC-1 were cultured in 5% CO2, 37 °C, RPMI Medium 1640 (Invitrogen) with 10% fetal bovine serum (FBS), Non-essential Amino Acids, Glutamax, and Sodium Pyruvate added. Medium and additional reagents were purchased from Invitrogen, FBS was purchased from Gibco. TRIzol (Lablead) was used to lysate and isolate RNA from cells in logarithmic growth phase according to the manufacturer’s protocol.

### Quantitative real-time polymerase chain reaction (RT-qPCR)

RT-qPCR was conducted after reverse transcription and performed essentially as described previously with housekeeper (GAPDH) mRNA for normalization via the 2^−ΔΔCt^ method [28]. Each experiment was repeated 3 times. Sequences of primers were listed as shown in Table 1.

### Gene set enrichment analysis (GSEA) of the 11-gene signature

We explored the potential molecular alterations of the signature by GSEA [29]. 488 PTC samples from the TCGA-THCA dataset were defined as low- or high-risk by the optimal cut-off value generated by X-Tile [30]. GSEA v4.2 has then applied to find the biological alteration in the high-risk group. The gene sets included C2: KEGG [24], C5: GO, and C6: oncogenic signatures. FDR < 0.05 with |NES| > 1 were considered to indicate significant enrichment.

### Independent prognostic parameters in PTC

We performed uni- and multivariate Cox analyses to find the correlated prognostic parameters in PTC. Clinical parameters included age, gender, ethnicity, BRAFV600E mutation, RAS mutation, extrathyroidal extension, neoplasm size, histological type, anatomic sites of tumors, residual tumor and disease TNM stage. The univariate analysis was performed first, then the factors with p < 0.2 were enrolled in multivariate analysis to identify independent ones. A p-value of < 0.05 is statistically significant.

### Construction of the novel nomogram

After the collinearity diagnosis, a novel stepwise Cox regression model incorporating independent and relevant clinical factors was built and visualized as a nomogram for predicting the 1-, 3-, and 5-year PFI survival of PTC. The length of each parameter stands for its” weight in regression model. We then evaluated the nomogram’s predictive power with the ROC curve, C-index, calibration curve and decision curve analysis (DCA) [31]. The calibration curve was generated by a bootstrap method with 1000 resamples.

### Statistical analysis

We used R v3.6.3 and GraphPad Prism 8.4.3 (GraphPad Software, U.S.) for statistical analysis. Categorical variables were analyzed using Chi-squared test. Normality of continuous data was analyzed using Shapiro-Wilk test. Continuous data were analyzed using unpaired t-test or Mann Whitney test. A p-value of < 0.05 was considered statistically significant.

## Results

### DE-GaGs identification and GEO datasets acquisition

**Fig. 1:**
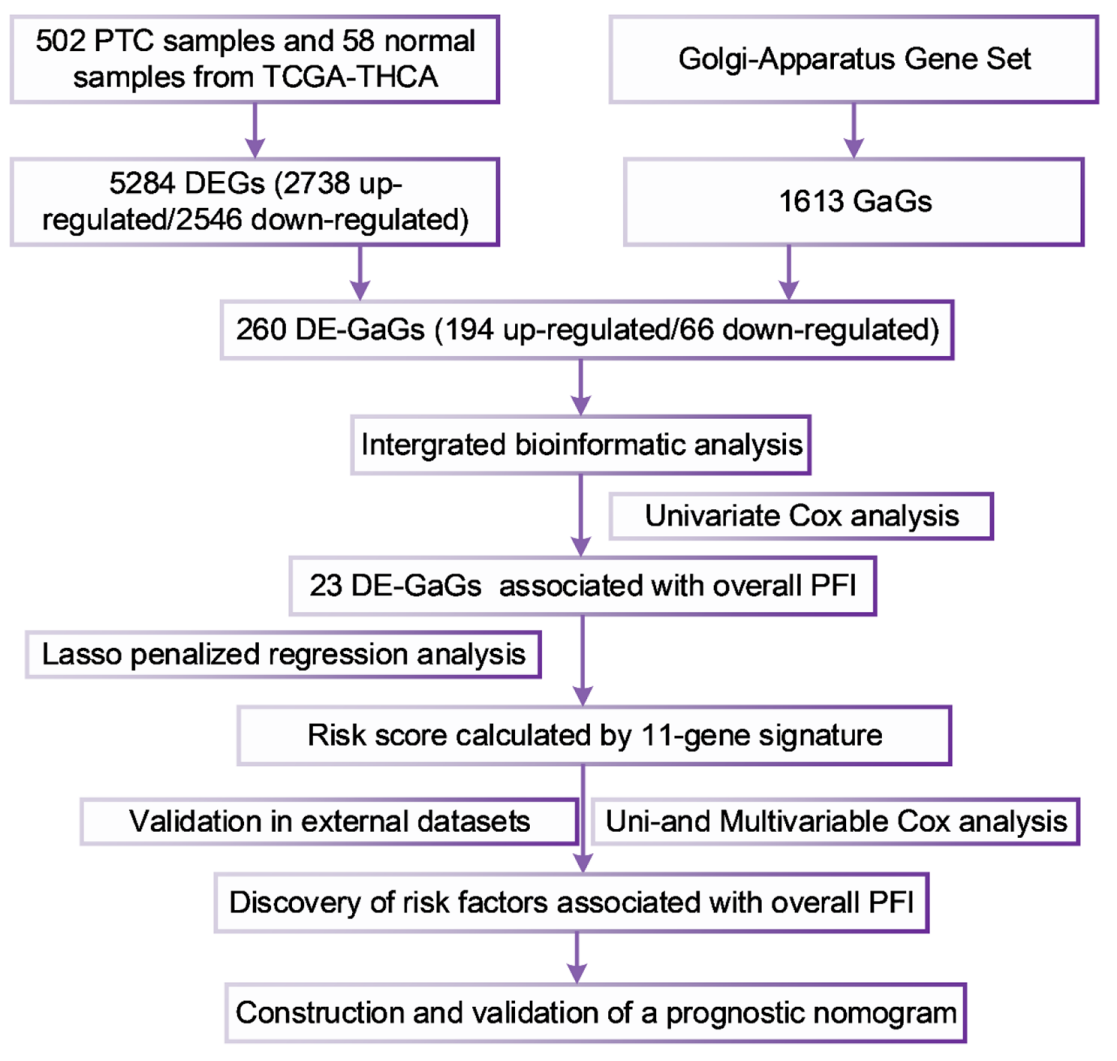
Flowchart describing the process of establishment, optimization and validation of the novel 11-gene signature and prognostic nomogram

**Fig. 2:**
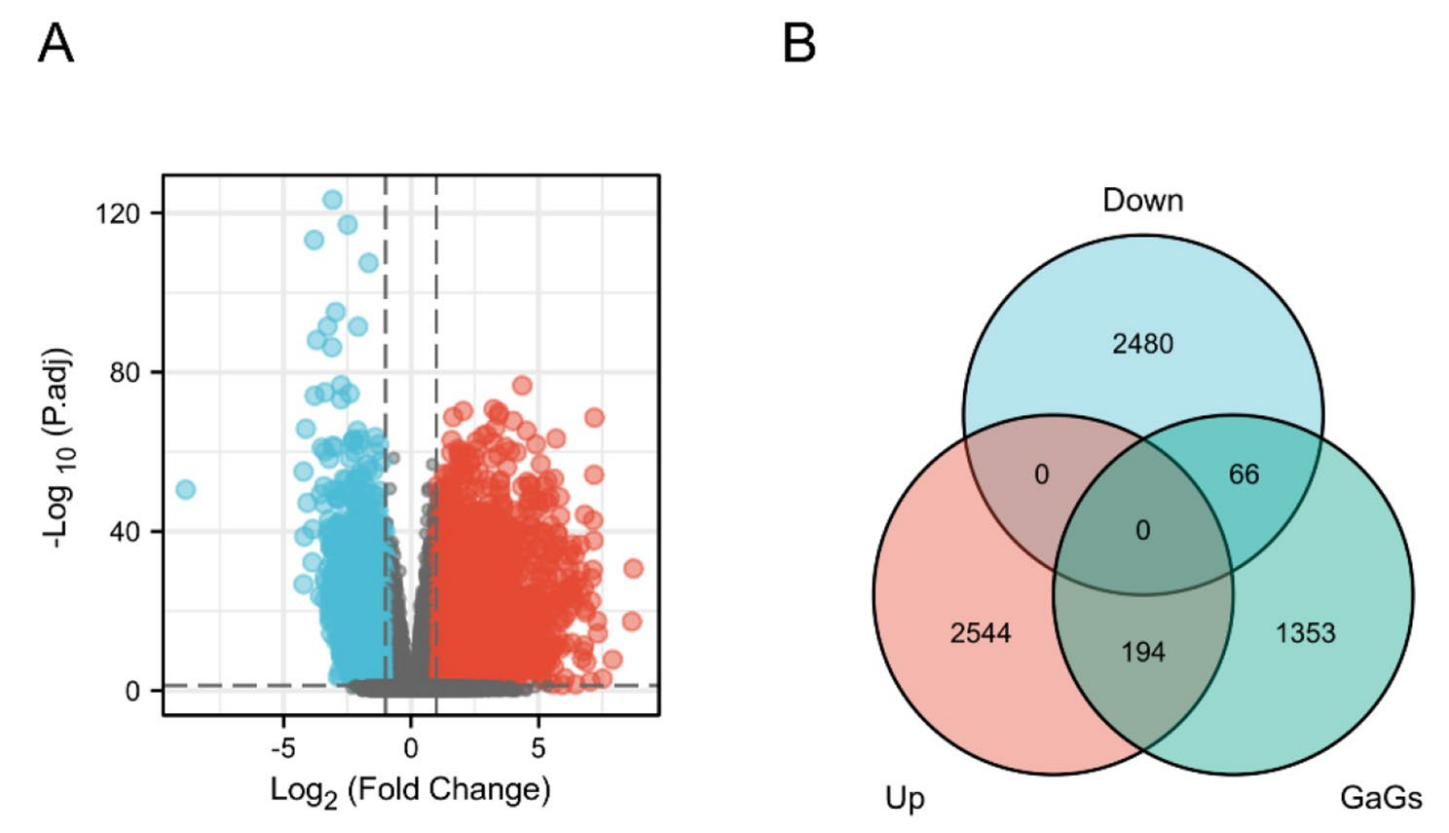
Identification of DE-GaGs in PTC. (A) Volcano map presenting the 5,284 DEGs (2,738 up- and 2,546 downregulated) in PTC. (B) 260 DE-GaGs (194 up and 66 downregulated) were identified based on the intersection

Figure 1 indicated the main methodological process of the study. We enrolled 488 PTC cases using total follow-up information (follow-up days longer than 30 days) for our analysis. Volcano plot showed the identification of 5,284 DEGs (2,738 up- and 2,546 downregulated) (Fig. 2A). Furthermore, we downloaded the list of 1,613 GaGs from the golgi apparatus-related gene set on the Molecular Signatures Database (https://www.gsea-msigdb.org/gsea/msigdb/index.jsp). And we made an intersection between the 1,613 GaGs and the 5,284 DEGs (2,738 up- and 2,546 downregulated), then the intersection contained 260 DE-GaGs (194 up and 66 downregulated) (Fig. 2B). Supplementary Tables 1 and 2 presented the full list of the 1,613 GaGs and the 260 DE-GaGs. After that, we enrolled 3 datasets focused on PDTC or ATC: GSE29265 contributed by Tomas G, et, al (20PTCs, 20Normals, 9ATCs), GSE33630 [32] (49PTCs, 45Normals, 11ATCs) and GSE76039 [25] (20ATCs, 17PDTCs).

### Functional enrichment analysis of the 260 DE-GaGs

**Fig. 3:**
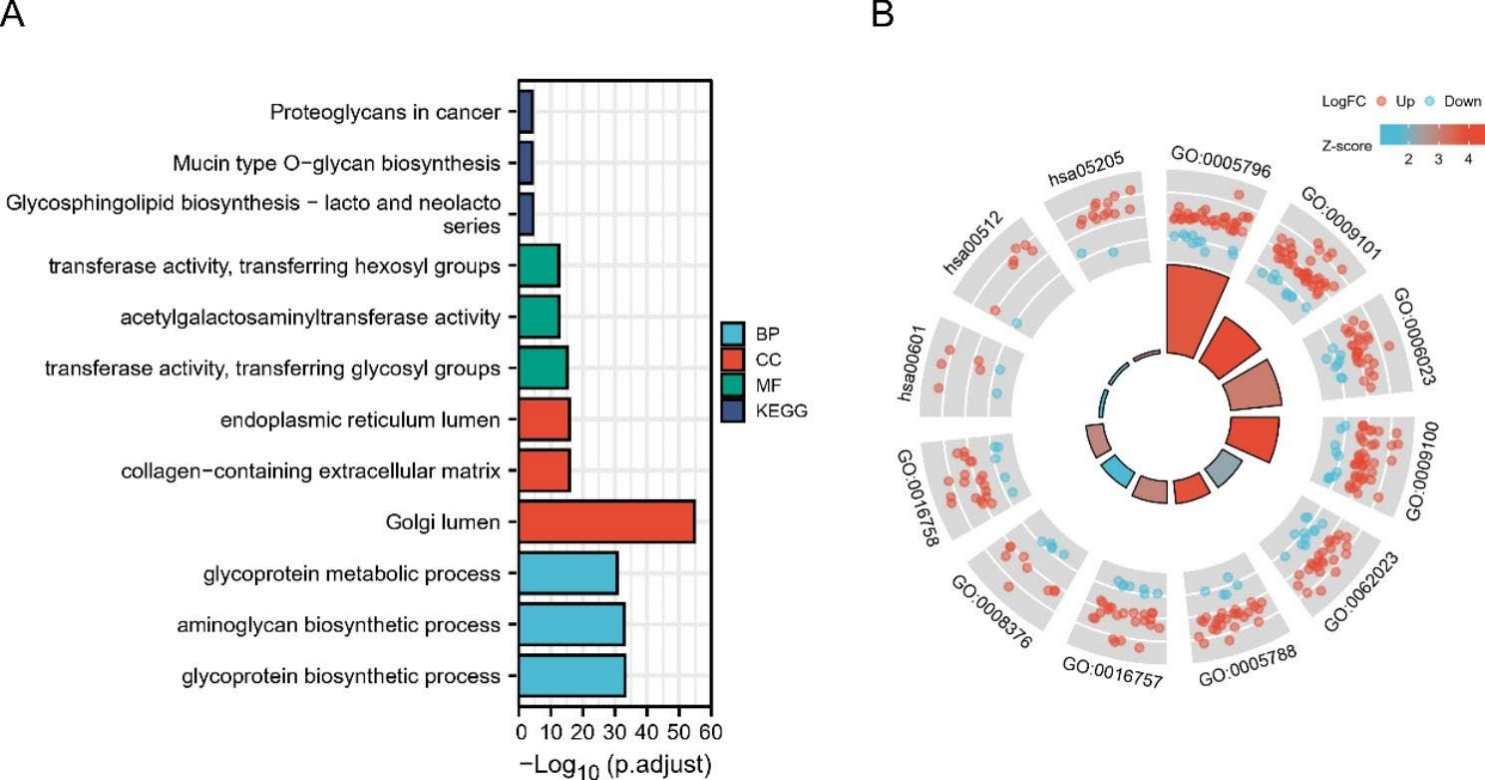
Functional enrichment analysis of the 260 DE-GaGs. (A) The top 20 enriched gene ontology (GO), biological process (BP), cellular components (CC) and the molecular function (MF) terms of the DE-GaGs [21]. (B) The enriched terms in combination of each value of LogFC of the DE-GaGs.

Annotation of the 260 DE-GaGs by GO and KEGG pathway analyses is shown in Fig. 3. For the biological process (BP) category, the DE-GaGs were mainly enriched in glycoprotein biosynthetic, aminoglycan biosynthetic, and glycoprotein metabolic processes. In terms of the cellular component (CC) category, the DE-GaGs were mainly enriched in the endoplasmic reticulum lumen, collage-containing extracellular matrix, and golgi lumen. In the molecular function (MF) category, the DE-GaGs were mainly enriched in transferase activity, transferring glycosyl groups, acetylgalactosaminyltransferase activity, and transferase activity, transferring hexosyl groups. Regarding KEGG pathways, the DE-GaGs were mainly enriched in Proteoglycans in cancer, Mucin type O-glycan biosynthesis, and Glycosphingolipid biosynthesis-globo and isoglobo series (Fig. 3A). Enrichment results accompanied with respective Z-scores and LogFC were as shown in Fig. 3B. A full list of functional enrichment results was listed in supplementary Table 3.

**Table 1:** Sequences of primers for RT-qPCR analyses

Target gene	Primer	Sequence (5'-3')
GAPDH	forward	AGTCCCTGCCACACTCAG
	reverse	TACTTTATTGATGGTACATGACAAGG
POSTN	forward	CACCAATGAGGCTTTTGAGAAA
	reverse	GACTGCTCCTCCCATAATAGAC
KIF20A	forward	GAATGTGGAGACCCTTGTTCTA
	reverse	CCATCTCCTTCACAGTTAGGTT
ATG9B	forward	TGCCAACCAACCAAGTAACCATACC
	reverse	CACTGGGCTGAGGGTAGGATGG
RNF144A	forward	GTGCCTGAAACAGTATGTTGAG
	reverse	CAAACAGCACCTCTCTTTCAAA
TMEM130	forward	GCAGGAAACCCTTCGAGGCATC
	reverse	CAGGAAGTTCAAGGTCACGGTCATC
PKMYT1	forward	CTGTGTGGAGCAAAGAGGTTTC
	reverse	TGTTAATGACCATACAAACGCC
MANEAL	forward	CGTCCTGGTCCTGTCCTGGTAC
	reverse	GCCACCTGGATGCTGTACTGATG
CAPAN8	forward	GGCGGAAGGAAGAACTGGACAAG
	reverse	CCGAGAGAACTGCCTCACGAAATC
ABCA12	forward	AGAACAATCATTCTGTCAACGC
	reverse	GGAGATGTGATTGGATCATTGC
SOD3	forward	GGAGTGGATCCGAGACATGTA
	reverse	CGAAGAAGGCGTCGAGCTT
SPRR3	forward	CTACACCAAGGTCCCTGAAC
	reverse	ACAGGAACTTTGGTGTATCCTT

### Screening of significant DE-GaGs and construction of the novel 11-gene signature

**Fig. 4:**
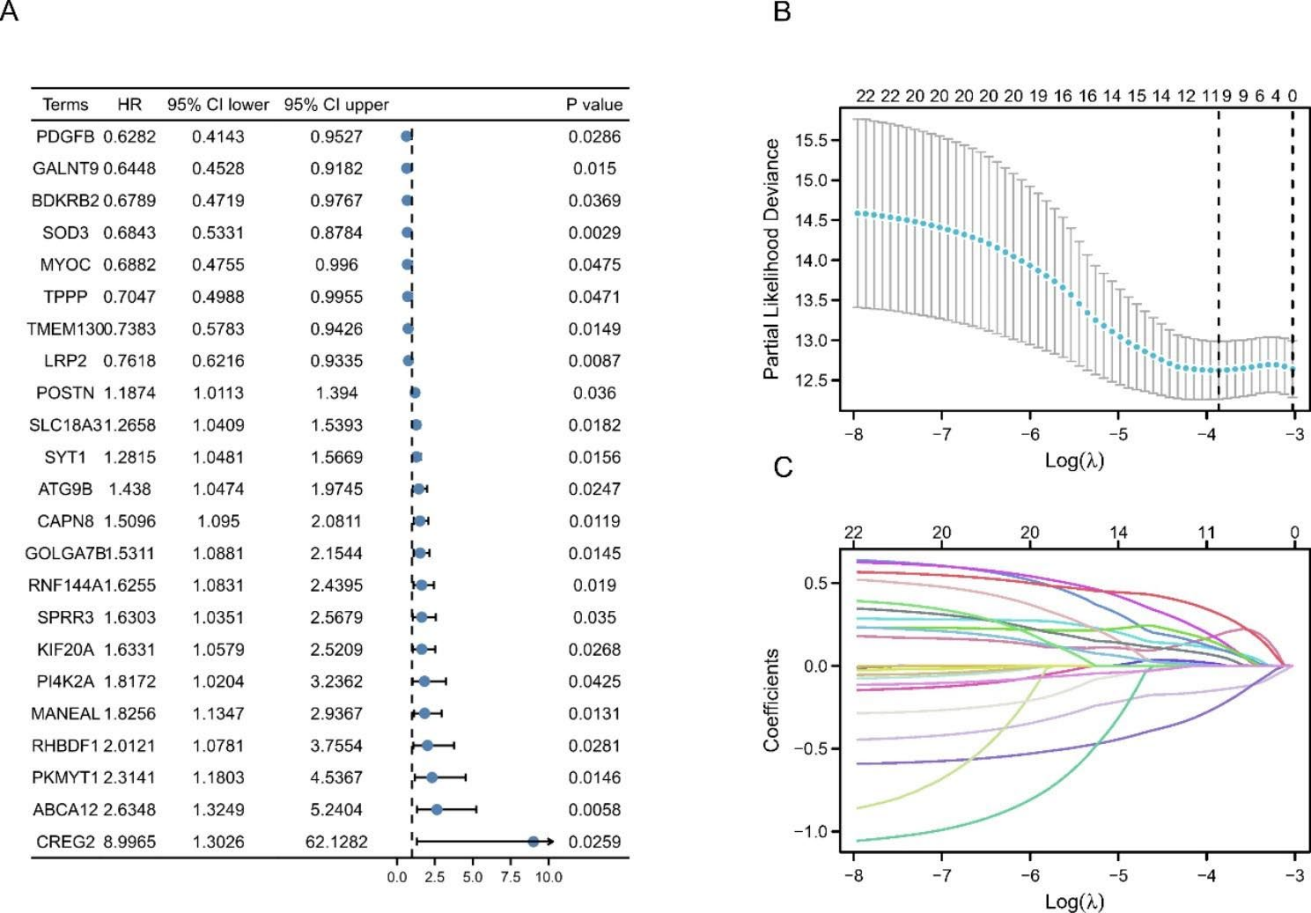
Differential expression level and hazard ratios (HR) of the 23 DE-GaGs in training set. (A) Forest plot with hazard ratios (HR) representing the predictive values of the 23 DE-GaGs that were PFI-related in PTC. (B) LASSO coefficient profiles of the 23 DE-GaGs. (C) Lasso deviance profiles of the 23 DE-GaGs. The lambda selection criterion was based on the value of lambda giving a minimum mean cross-validation error

Table 2 shows the baseline information of the training and testing sets allocated from 488 PTC cases. In total, 23 DE-GaGs related to PFI were identified. Figure 4 A shows forest plots of each item’s logfc, P-value and hazard ratio. LASSO penalty regression analyses reduced and constructed a novel 11 gene signature, as shown in Fig. 4B, C. The GaGs signature risk score was calculated as follows: risk score = exp/SOD3 ⋅ (-0.145442888) + exp/ABCA12 ⋅ 0.187674472 + exp/CAPN8 ⋅ 0.009918277 + exp/MANEAL ⋅ 0.098075693 + exp/PKMYT1 ⋅ 0.101879373 + exp/TMEM130 ⋅ (-0.243464188) + exp/RNF144A ⋅ 0.158935422 + exp/ATG9B ⋅ 0.313666103 + exp/KIF20A ⋅ 0.131711125 + exp/SPRR3 ⋅ 0.057542545 + exp/POSTN ⋅ 0.000852346.

**Table 2:** Baseline characters of 488 TCGA-THCA patients

Characteristic			Training			Testing		p		
n			381			107				
Progression, n (%)								1.000		
free			343 (70.3%)			96 (19.7%)				
progression			38 (7.8%)			11 (2.3%)				
RAS_status, n (%)								1.000		
Mutated			46 (9.4%)			13 (2.7%)				
Wild type			335 (68.6%)			94 (19.3%)				
BRAF_status, n (%)								0.958		
Mutated			217 (44.5%)			60 (12.3%)				
Wild type			164 (33.6%)			47 (9.6%)				
Extrathyroid_extension, n (%)								0.642		
Minimal (T3)			102 (21.7%)			28 (5.9%)				
Moderate/Advanced (T4)			16 (3.4%)			2 (0.4%)				
None			253 (53.7%)			70 (14.9%)				
Histological_type, n (%)								0.918		
Classical/usual			274 (56.1%)			77 (15.8%)				
Follicular			78 (16%)			23 (4.7%)				
Tall Cell			29 (5.9%)			7 (1.4%)				
Neoplasm_focus_type, n (%)								0.562		
Multifocal			167 (34.9%)			51 (10.7%)				
Unifocal			206 (43.1%)			54 (11.3%)				
Anatomic_site, n (%)								0.861		
Bilateral			62 (12.9%)			19 (3.9%)				
Isthmus			18 (3.7%)			4 (0.8%)				
Unilateral			298 (61.8%)			81 (16.8%)				
Residual_tumor, n (%)								0.122		
R0			296 (69.5%)			75 (17.6%)				
R1			35 (8.2%)			16 (3.8%)				
R2			4 (0.9%)			0 (0%)				
Ajcc_stage, n (%)								0.083		
Stage I			221 (45.5%)			52 (10.7%)				
Stage II			34 (7%)			17 (3.5%)				
Stage III			82 (16.9%)			28 (5.8%)				
Stage IV			43 (8.8%)			9 (1.9%)				
M_stage, n (%)								1.000		
M0			373 (76.6%)			106 (21.8%)				
M1			7 (1.4%)			1 (0.2%)				
N_stage, n (%)								0.677		
N0			179 (40.9%)			46 (10.5%)				
N1			165 (37.7%)			48 (11%)				
T_stage, n (%)								0.496		
T1			111 (22.8%)			30 (6.2%)				
T2			126 (25.9%)			35 (7.2%)				
T3			123 (25.3%)			40 (8.2%)				
T4			19 (3.9%)			2 (0.4%)				
Gender, n (%)								0.620		
Female			277 (56.8%)			81 (16.6%)				
Male			104 (21.3%)			26 (5.3%)				
Age, n (%)								0.860		
< 55			255 (52.3%)			70 (14.3%)				
≥ 55			126 (25.8%)			37 (7.6%)				
Progression_free_interval, meidan (IQR)			938 (491, 1463)			678 (509, 1259)		0.195		

**Verification of the discriminatory power of the novel 11 gene signature.**

**Fig. 5:**
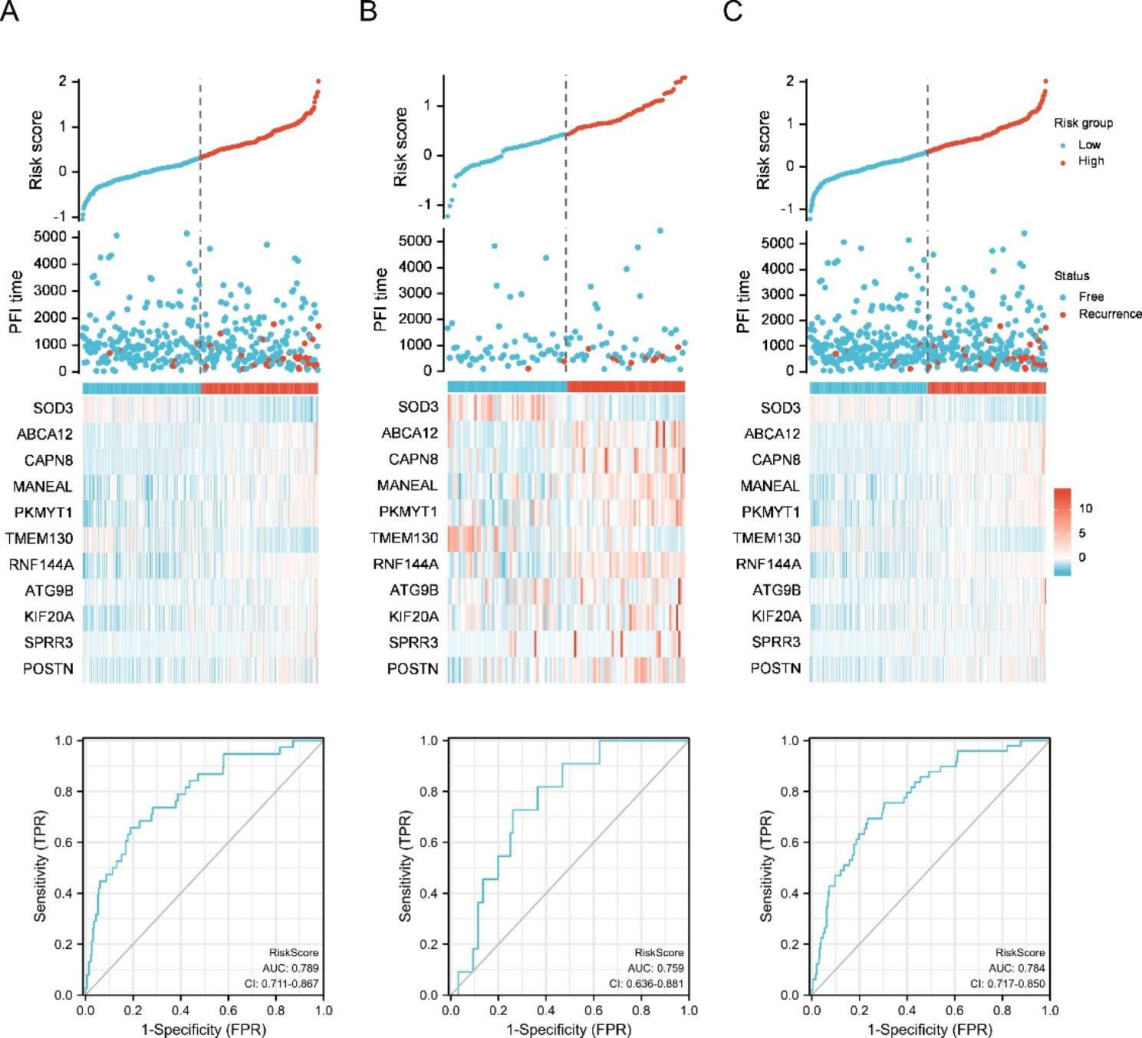
Evaluation of the efficacy of the 11-gene signature in the TCGA-THCA dataset. The dataset was randomly divided into the training set, and the validation set with a 4/5 ratio. (A-C) Relationship between the signature risk-scores (up) and recurrence status of patients of high/low-risk (middle) in training/validation/total TCGA-THCA dataset. Time-dependent ROC for the predictions of PFI for the 11-gene signature in the training/validation/total sets

Figure 5 A-C shows the relationships between signature risk score and recurrence events as scatter plots. In the training set, the AUC for PFI prediction based on the 11 gene signature was 0.789 (95% CI 0.711–0.867). In the testing set, the AUC was 0.759 (95% CI 0.636–0.881). In the TCHA total dataset, the AUC was 0.784 (95% CI 0.717–0.850). The optimal cut-off value for discriminating high-risk patients of PTC on Illumina Hiseq 2500 platform was 1.06 according to X-Tile software [30].

### Clinical correlation and verification of the GaG-signature

**Fig. 6:**
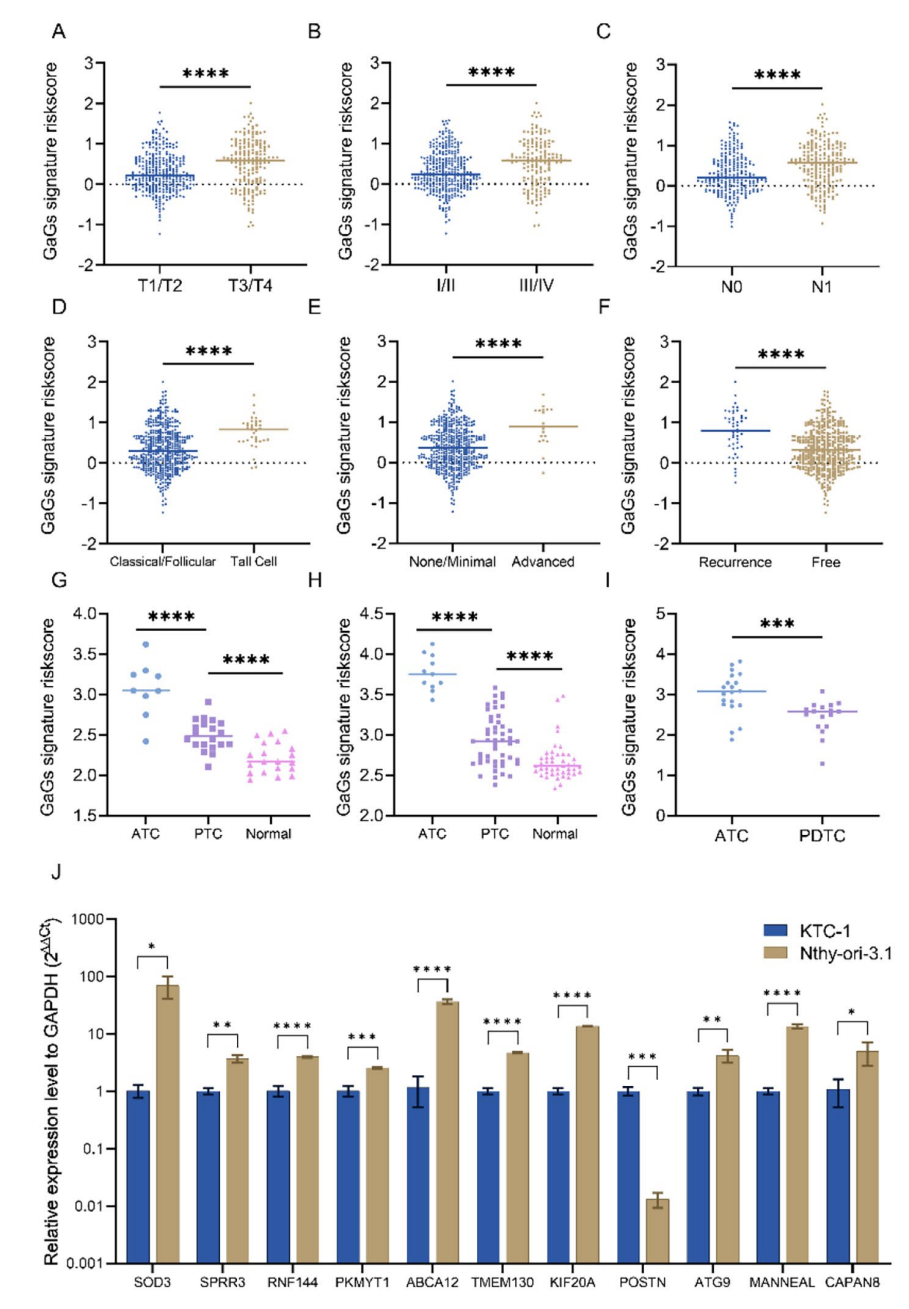
Correlations between GaGs signature with clinical or biological characters in TGCA-THCA cohort, GEO datasets and PTC cell lines. (A-F) The distribution of the signature risk-scores according to different status of T stage, disease stage, lymph node metastasis, histological type, extrathyroidal invasion and progression status in the TCGA-THCA dataset. (G-I) The distribution of the signature risk-scores according to different type of tumor tissues from three external datasets. Papillary thyroid carcinoma (PTC), anaplastic thyroid carcinoma (ATC) and poorly differentiated thyroid carcinoma (PDTC). (J) Relative expression level of 11 Golgi signature genes to GAPDH (2^−ΔΔCT^) in Nthy-ori 3.1 and KTC-1 cell line (n = 3). Data are presented as interleaved bar plot. Scale of left Y axis was presented as log 10 format. Unpaired t test or Mann-Whitney test, *P < 0.05 ***P < 0.001

Next, we analyzed the correlation between the GaG-signature and clinical characters after normality test. In groups divided by T stage, patients in T1/T2 had a lower signature risk score than those in an advanced disease stage (T3/T4) (Fig. 6A). Patients in stage 1/2 had lower signature risk score than those in stage 3/4 (Fig. 6B). Patients in N1 had higher signature risk score than those without lymphnode metastases (N0) (Fig. 6C). Patients with aggressive histological (tall cell) type have higher signature risk score than those with non-aggressive histological type (Fig. 6D). In groups divided by residual tumor, patients without residual tumor had a lower signature risk score than those with residual tumor (Fig. 6E). In groups divided by recurrence status, patients without recurrence had lower signature risk scores than patients with disease progression (Fig. 6F). The differences were statistically significant (P < 0.05). We also validated the pattern of GaG-signature in 3 external GEO datasets and compared the signature risk score between ATC/PDTC/PTC. In the datasets, GSE 29,265 and GSE 33,630, signature risk scores were higher in ATC samples than PTC samples (p < 0.0001, respectively), as shown in Fig. 6G, H. In the dataset GSE 76,039, signature risk scores were higher in ATC samples than in PDTC samples (p < 0.001), as shown in Fig. 6I.

### RT-qPCR quantification of GaG-signatures in PTC cell lines

The relative 11 gene expression level of Golgi signature in Nthy-ori 3.1 and KTC-1 cell were generated through RT-qPCR quantification. In the 11 genes of Golgi signature, the expression level of POSTN was higher in KTC-1 than Nthy-ori 3.1, while SOD3, SPRR3, RNF144A, PKMYT1, ABCA12, TMEM130, KIF20A, ATG9, MANEAL and CAPN8 were lower in KTC-1 than Nthy-ori 3.1, the differences were statistically significant (P < 0.05), as shown in Fig. 6J.

#### GSEA

**Fig. 7:**
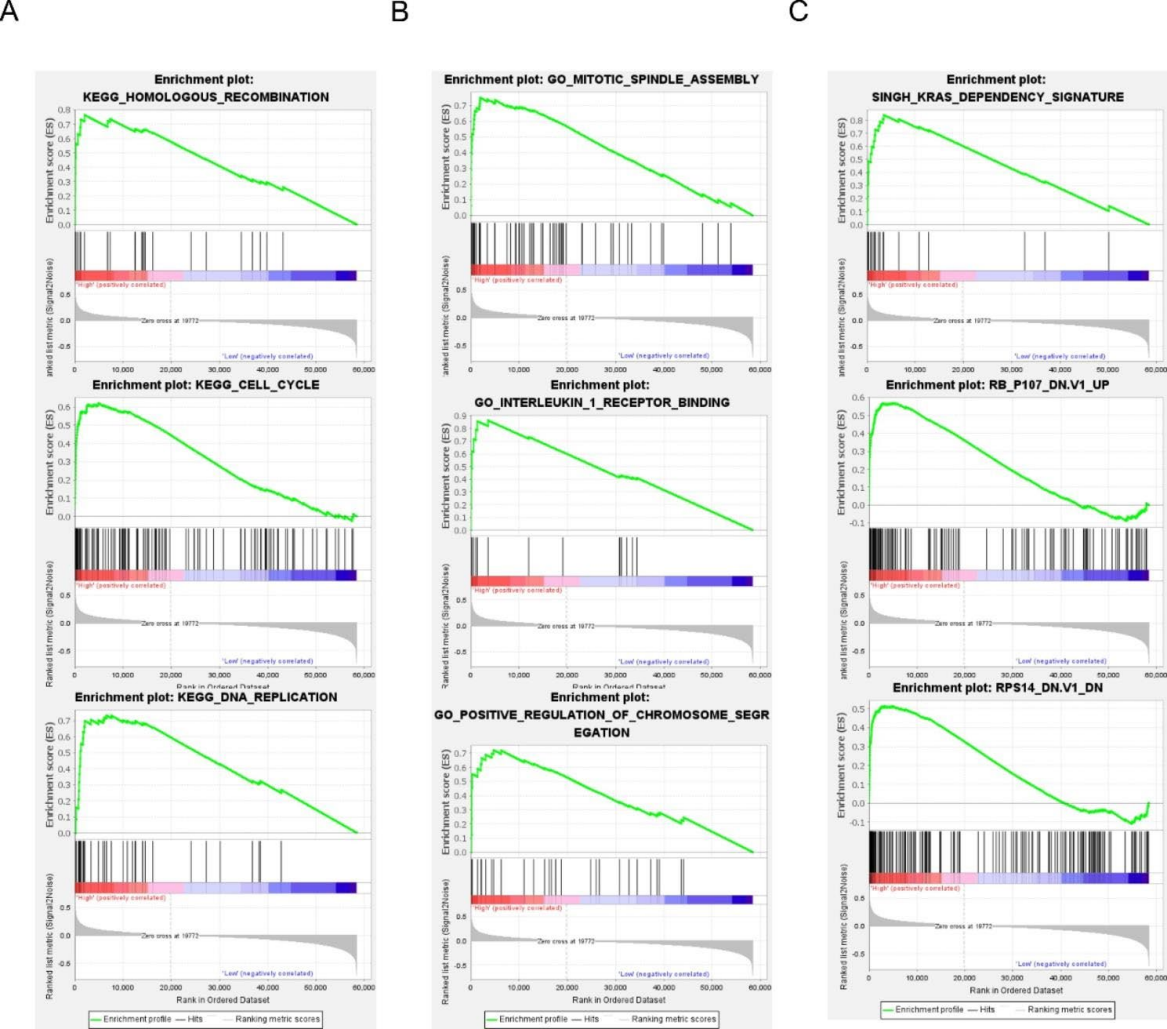
Gene set enrichment analysis (GSEA) analysis of the 11 gene signature. (A-C) Representative signaling pathways, biological functions, and oncogenic signatures significantly enriched in the high-risk group identified by GSEA.

GSEA in the 488 PTC cases from the THCA dataset showed the representative altered biological functions of the high-risk group (Fig. 7A-C). For KEGG pathways, the molecular alterations in the high-risk group samples were related to the homologous recombination, cell cycle, and DNA replication. For the c5 gene ontology terms, the molecular alterations were related to mitotic spindle assembly. For the oncological signatures, the alterations included the KRAS dependency signature. Detailed GSEA results were presented in Supplementary Table 4.

**Identification of independent prognosis-related parameters.**

The univariate Cox analysis revealed that parameters including age (≥ 55), neoplasm size (≥ 2 cm), histological type (tall cell), T stage (T3/T4), M stage (M1), TNM stage and signature risk scores were significantly associated with prognosis (P < 0.05). After the exclusion of samples with incomplete information and P < 0.2 in univariate analysis, a total of 408 patients were enrolled in multivariate analysis. In multivariate analysis, neoplasm size (≥ 2 cm) and signature risk scores were significantly associated with prognosis (P < 0.05), which were then identified as independent prognosis-related factors in both the uni- and multivariate analysis as shown in Table 3.

**Table 3:** Uni- and multivariate analysis for identification of prognostic factors

Characteristics	Total(N)	Univariate analysis		Multivariate analysis	
		Hazard ratio (95% CI)	P value	Hazard ratio (95% CI)	P value
Riskscore	488	4.531 (2.689–7.637)	**< 0.001**	3.038 (1.648–5.598)	**< 0.001**
Ethnicity	388				
Not Hispanic or Latino	354	Reference			
Hispanic or Latino	34	0.463 (0.112–1.918)	0.289		
BRAF status	488				
Wild type	211	Reference			
Mutated	277	1.455 (0.801–2.645)	0.218		
RAS status	488				
Wild type	429	Reference			
Mutated	59	1.640 (0.768–3.504)	0.201		
Extrathyroid extension	471				
Moderate/Advanced (T4)	18	Reference			
None/Minimal	453	0.476 (0.171–1.326)	0.156	1.609 (0.457–5.668)	0.459
Neoplasm size	474				
< 2cm	153	Reference			
≥ 2cm	321	3.914 (1.547–9.901)	**0.004**	2.729 (1.040–7.159)	**0.041**
Histological type	488				
Classical/Follicular	452	Reference			
Tall Cell	36	2.417 (1.084–5.389)	**0.031**	1.305 (0.508–3.348)	0.580
Anatomic site	482				
Unilateral	379	Reference			
Bilateral	81	1.101 (0.513–2.365)	0.805		
Isthmus	22	0.413 (0.057–3.004)	0.382		
M stage	487				
M0	479	Reference			
M1	8	5.630 (2.021–15.687)	**< 0.001**	1.422 (0.380–5.323)	0.601
Residual tumor	426				
R0/R1	422	Reference			
R2	4	2.027 (0.277–14.813)	0.486		
Ajcc_stage	486				
Stage III/IV	162	Reference			
Stage I/II	324	0.363 (0.207–0.638)	**< 0.001**	0.717 (0.279–1.845)	0.491
N stage	438				
N0	225	Reference			
N1	213	1.736 (0.950–3.172)	0.073	1.114 (0.559–2.218)	0.759
T stage	486				
T1/T2	302	Reference			
T3/T4	184	2.806 (1.569–5.018)	**< 0.001**	1.151 (0.539–2.458)	0.716
Gender	488				
FEMALE	358	Reference			
MALE	130	1.747 (0.977–3.124)	0.060	1.322 (0.697–2.509)	0.393
Age	488				
≥ 55	163	Reference			
< 55	325	0.443 (0.252–0.776)	**0.004**	0.675 (0.291–1.567)	0.360

### Establishment and validation of the novel signature-based nomogram

**Fig. 8:**
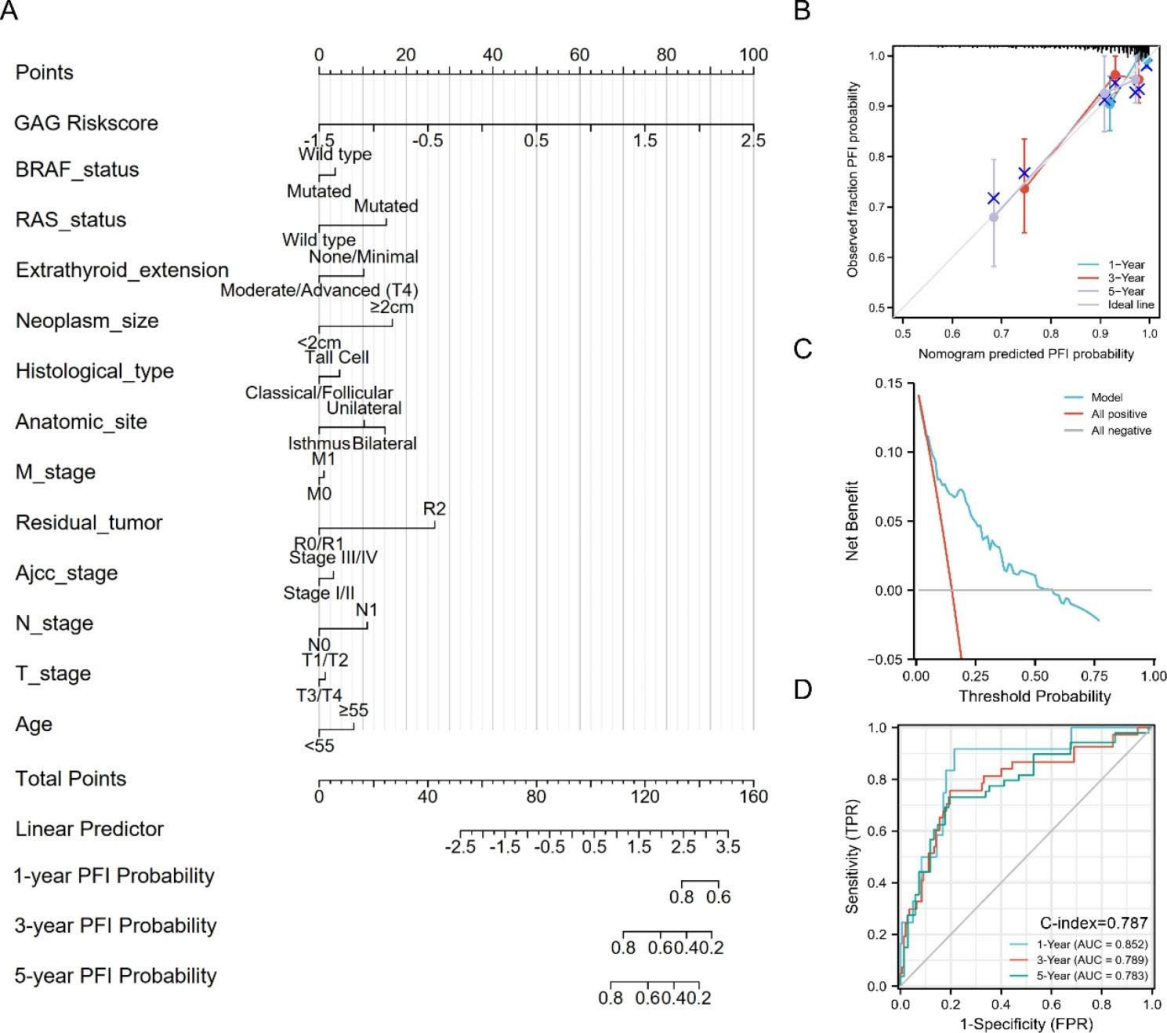
Construction and validation of the nomogram in predicting PFI of PTC in the TCGA-THCA dataset. (A) A nomogram based on the 11-gene signature and relevant clinical features for forecasting the PFI of PTC. (B) The calibration curve for internal validation of the nomogram. (C) The DCA curve showing the clinical utility of the nomogram in 5-year PFI. (D) Time-dependent ROC for predicting the 1-, 3- and 5-year PFI of PTC.

We constructed a stepwise Cox regression model including riskscore, age, TNM stage, neoplasm size, residual tumor, histological type and RAS status. The model was visualized in a predictive nomogram, as shown in Fig. 8A. Evaluation of the predictive nomogram using the calibration curve and decision curve revealed the efficacy and robustness of the model for the prediction of the prognosis of PTC patients (Fig. 8B, C). The AUCs for 1-year, 3-year, and 5-year PFI predictions were 0.852, 0.789, and 0.783, respectively, with a C-index of 0.787 (Fig. 8D).

## Discussion

Most patients with PTC achieve a relatively good prognosis. However, persistent disease or recurrences are observed in 5-20% of patients, associated with severe complications following re-operation or other therapies [33]. For patients with a low risk of recurrence, prolonged thyroid-stimulating hormone suppression therapy may cause multiple adverse effects such as osteoporosis, osteopenia and atrial fibrillation [34]. Considering the relatively excellent prognosis, developing novel diagnostic tools with high sensitivity and specificity seems to have greater clinical significance than exploring neoadjuvant therapies. Traditional staging systems such as the ATA risk stratification system allow evaluation of recurrence risk with a stratified population rather than individualized risk, which indicates that a group of patients sharing the same clinical and pathological characteristics would have the same chance of recurrence [35]. However, the biological mechanisms underlying PTC progression are highly complex and heterogeneous, requires more accurate and personalized prediction models at the molecular level. Therefore, specified gene signatures would effectively predict the metastatic and recurrent potential tumors.

The incidence of PTCs has been continually increasing; however, the mortality rate has not changed substantially, which is may because most PTCs diagnosed incidentally are low-risk papillary thyroid microcarcinomas (PTMCs). Active surveillance appears to be safe except for tumors with high-risk features such as extrathyroidal extension, clinically evident LNM(+), and particular aggressive types [36]. It can replace immediate surgery for low-risk PTC [37]. Active surveillance begins when patients are diagnosed with low-risk PTC by ultrasound examination of fine-needle aspiration biopsy (FNAB). Since PTCs involve complex biological mechanisms, the decision to perform active surveillance based on genomic signatures followed by FNAB with a micro-assessing technique such as droplet digital PCR [38] (ddPCR) would be safer than assessments based on superficial clinical or imaging characteristics. Therefore, patients with a higher risk score but with a low risk of clinical features would be treated more rationally.

Multiple shreds of evidence showed the pivotal role of malfunctioned Golgi apparatus gadgets in thyroid cancer invasion and progression [13, 39, 40]. Considering the main function of Golgi apparatus of processing, sorting, and then transporting the proteins to specific parts of cells or secreting them outside [41], combined with the fact that cancer cells metabolize and grow vigorously to support its” invasion and metastasis, we assumed that the expression pattern of Golgi apparatus would be closely related with PTC prognosis, which motivated us to focus on differentially expressed GaGs derived from the MSigDB. Based on principles of machine learning, the contents of the training set should be as large as possible to be close to reality; then we applied the 4/5 ratio of training set instead of a 1:1 split and confirmed a satisfied AUC in a testing cohort. We reduced the variables from 260 to 23 DE-GaGs that were PFI-related. GO enrichment analysis showed that the 260 DE-GaGs were enriched in the glycoprotein biosynthetic process, aminoglycan biosynthetic process, and glycoprotein metabolic process, which, consistent with the definition of GaGs, have been proven to be associated with cancer metastasis [16, 41]. In the identified 11 genes, several were previously proved to be associated with PTC progression through experiments. For example, stromal SOD3 had a stimulatory effect on thyroid cancer cell growth and an inhibitory effect on cancer cell migration [42], POSTN expression was activated by ΔNp73 and modulated epithelial-mesenchymal transition of thyroid cancer cells [43]. Thus, these genes have the potential to predict metastasis and recurrence in PTC. However, the fundamental role of left nine components had not been studied, which would be new targets for thyroid cancer biology research.

Besides, we explored the potential molecular alteration by the 11-gene signature using GSEA. GSEA, which is based on careful consideration of all differential genes’ roles, can help reveal the complex behavior of genes in health and disease more accurately. In contrast, traditional strategies, including KEGG or GO, focus on identifying individual genes that exhibit differences [44]. Multiple gene expression alterations in the high-risk group were involved in tumor biology processes, such as homologous recombination and cell cycle pathways. Thus, the potential mechanisms underlying patients’ poor PFI in the high-risk group could be elucidated. Further, the signature’ ’s utility of differentiating normal from PTC samples also had been validated in multiple external datasets. In the aspects of the clinical correlation, we found that patients with advanced or worse clinical status, such as advanced extra-invasion existence, advanced TNM stages, residual tumor, and tall cell histological type, had higher signature risk scores, which strongly demonstrated the clinical efficacy of the GaGs signature. To our knowledge, patients with ATC and PDTC only have a mean survival after diagnosis of 0.5 and 3.2 years, respectively, and de-differentiation is a significant reason for the highly malignant degree [25]. The significantly higher gene risk scores in ATC samples could partly confirm our conjecture. Also, we applied qPCR experiment of 11 genes on PTC cell line KTC-1 and normal thyroid follicular cell line Nthy-ori-3.1. The KTC-1, which originated from advanced metastatic PTC and refractory to radio iodine therapy, was with low degree of differentiation and was highly invasive [27]. According to the results, the 2 major tumor suppressor genes (HR < 1 with negative coefficient in Golgi signature) (SOD3 and TMEM130) were expressed higher in Nthy-ori-3.1, also confirmed the connection between Golgi signature with PTC invasion and de-differentiation.

Nomograms are widely used for the ability to present the numerical probability of a particular clinical event by integrating prognostic variables [45]. Nomograms, including a risk score based on gene signatures and clinicopathological parameters, can predict prognosis more precisely after surgery [46, 47]. Moreover, numerical results are more comfortable for patients to understand than the traditional staging system. To the best of our knowledge, the GaGs-based signature and the relevant nomogram achieved the highest AUC for predicting PFI in the TCGA-THCA cohort and has not been reported yet. The limited number of genes made it more practical and economically feasible than whole-genome sequencing. Moreover, since the significantly worse clinical outcomes with PDTC/ATC than with PTC, the unique advantage in discovering the de-differentiation potential of TC made the 11-gene signature feasible in individualized follow-up.

There were limitations to our study. First, the primary source of RNA sequencing data and clinical information was the TCGA program, in which the source of samples was from North American people. When applying the model to patients from different countries or regions, possible deviations or biases would occur. Second, due to the lack of a large independent dataset of PTC with complete follow-up information, we validated the nomogram’s power on the TCGA dataset itself. We carried out experimental and GEO datasets validation. Future validation of external datasets with complete follow-up data is necessary. Last, since the signature was based on high-throughput sequencing data, the related cut-off point was suitable for data obtained from a similar platform but would not be directly applied in ddPCR results, which need further exploration in a large independent cohort.

## Conclusion

We built a novel Golgi apparatus related 11-gene signature, then established a nomogram combining the signature and relevant clinical and pathological factors for predicting PTC PFI. The efficacy of novel GaGs signature and relevant nomogram was satisfying, which achieved the best efficacy in the TCGA-THCA cohort as the best we know. It would be helpful for individualized active and postoperative surveillance strategies.

## Electronic supplementary material

Below is the link to the electronic supplementary material.

Supplementary Material 1

Supplementary Material 2

Supplementary Material 3

Supplementary Material 4

## Data Availability

We obtained all the datasets from the TCGA (https://portal.gdc.cancer.gov/), the UCSCxena (https://xenabrowser.net/datapages/), the MsigDB (https://www.gsea-msigdb.org/gsea/msigdb/index.jsp) and the Cbioportal database (http://www.cbioportal.org/). The databases are open accessed and available for the public.

## Abbreviations

AUC: Area under the curve
PTC: Papillary thyroid carcinoma
PTMCs: Papillary thyroid microcarcinomas
ATC: Anaplastic thyroid cancer
PDTC: Poorly differentiated thyroid cancer
GaGs: Golgi apparatus related genes
DE-GaGs: Differentially expressed GaGs
PFI: Progression-free interval
ROC: Receiver operating characteristic
C-index: Concordance index
GO: Gene Ontology
BPs: Biological processes
CCs: Cellular components
MFs: Molecular functions
KEGG: Kyoto Encyclopedia of Genes and Genomes
ATA: American Thyroid Association
GEO: Gene Expression Omnibus
TCGA: The Cancer Genome Atlas Program
ddPCR: Droplet digital PCR
AJCC: American Joint Committee on Cancer
LASSO: Least absolute shrinkage and selection operator

## References

[1] La Vecchia C, Malvezzi M, Bosetti C, Garavello W, Bertuccio P, Levi F (2015). Thyroid cancer mortality and incidence: a global overview. Int J Cancer.

[2] Rahib L, Smith BD, Aizenberg R, Rosenzweig AB, Fleshman JM, Matrisian LM (2014). Projecting Cancer incidence and deaths to 2030: the unexpected burden of thyroid, liver, and pancreas cancers in the United States. Cancer Res.

[3] Lim H, Devesa SS, Sosa JA, Check D, Kitahara CM (2017). Trends in thyroid Cancer incidence and mortality in the United States, 1974–2013. Jama-Journal of the American Medical Association.

[4] Bilimoria KY, Bentrem DJ, Ko CY, Stewart AK, Winchester DP, Talamonti MS (2007). Extent of surgery affects survival for papillary thyroid cancer. Ann Surg.

[5] Haugen BR, Alexander EK, Bible KC, Doherty GM, Mandel SJ, Nikiforov YE (2016). 2015 american thyroid Association Management Guidelines for adult patients with thyroid nodules and differentiated thyroid Cancer: the american thyroid Association Guidelines Task Force on thyroid nodules and differentiated thyroid Cancer. Thyroid.

[6] Lee SG, Lee WK, Lee HS, Moon J, Lee CR, Kang SW (2017). Practical performance of the 2015 american thyroid Association Guidelines for Predicting Tumor recurrence in patients with papillary thyroid Cancer in South Korea. Thyroid.

[7] McLeod DSA, Zhang L, Durante C, Cooper DS (2019). Contemporary debates in adult papillary thyroid Cancer Management. Endocr Rev.

[8] Wu M, Li X, Zhang T, Liu Z, Zhao Y (2019). Identification of a nine-gene signature and establishment of a Prognostic Nomogram Predicting overall survival of pancreatic Cancer. Front Oncol.

[9] Du Y, Gao Y (2020). Development and validation of a novel pseudogene pair-based prognostic signature for prediction of overall survival in patients with hepatocellular carcinoma. BMC Cancer.

[10] Wu M, Yuan H, Li X, Liao Q, Liu Z (2019). Identification of a five-gene signature and establishment of a Prognostic Nomogram to predict progression-free interval of papillary thyroid carcinoma. Front Endocrinol (Lausanne).

[11] Lin P, Guo Y, Shi L, Li X, Yang H, He Y (2019). Development of a prognostic index based on an immunogenomic landscape analysis of papillary thyroid cancer. Aging.

[12] Liu J, Huang Y, Li T, Jiang Z, Zeng L, Hu Z (2021). The role of the golgi apparatus in disease (review). Int J Mol Med.

[13] Huang DH, Jin L, Xie WW, Lin Q, Chen X (2019). [Clinicopathological significance of golgi phosphoprotein 3 expression in papillary thyroid carcinoma]. Zhonghua Yi Xue Za Zhi.

[14] Zhao J, Yang C, Guo S, Wu Y (2015). GM130 regulates epithelial-to-mesenchymal transition and invasion of gastric cancer cells via snail. Int J Clin Exp Pathol.

[15] Tokuda E, Itoh T, Hasegawa J, Ijuin T, Takeuchi Y, Irino Y (2014). Phosphatidylinositol 4-phosphate in the golgi apparatus regulates cell-cell adhesion and invasive cell migration in human breast cancer. Cancer Res.

[16] Petrosyan A, Holzapfel MS, Muirhead DE, Cheng P-W (2014). Restoration of compact golgi morphology in advanced prostate cancer enhances susceptibility to galectin-1-induced apoptosis by modifying mucin O-glycan synthesis. Mol Cancer Res.

[17] Liberzon A, Subramanian A, Pinchback R, Thorvaldsdóttir H, Tamayo P, Mesirov JP (2011). Molecular signatures database (MSigDB) 3.0. Bioinformatics.

[18] Robinson MD, McCarthy DJ, Smyth GK (2010). edgeR: a Bioconductor package for differential expression analysis of digital gene expression data. Bioinformatics.

[19] Bolstad BM, Irizarry RA, Astrand M, Speed TP (2003). A comparison of normalization methods for high density oligonucleotide array data based on variance and bias. Bioinformatics.

[20] Yu G, Wang L-G, Han Y, He Q-Y (2012). clusterProfiler: an R package for comparing biological themes among gene clusters. OMICS.

[21] Kanehisa M, Goto S (2000). KEGG: kyoto encyclopedia of genes and genomes. Nucleic Acids Res.

[22] Rebsamen M, Knecht U, Reyes M, Wiest R, Meier R, McKinley R (2019). Divide and conquer: stratifying Training Data by Tumor Grade improves deep learning-based brain tumor segmentation. Front Neurosci.

[23] Friedman J, Hastie T, Tibshirani R (2010). Regularization Paths for generalized Linear Models via Coordinate Descent. J Stat Softw.

[24] Schröder MS, Culhane AC, Quackenbush J, Haibe-Kains B (2011). Survcomp: an R/Bioconductor package for performance assessment and comparison of survival models. Bioinformatics.

[25] Landa I, Ibrahimpasic T, Boucai L, Sinha R, Knauf JA, Shah RH (2016). Genomic and transcriptomic hallmarks of poorly differentiated and anaplastic thyroid cancers. J Clin Invest.

[26] Kim M, Kim S-J, Xu Z, Ha SY, Byeon JH, Kang EJ (2020). BRAFV600E transduction of an SV40-Immortalized normal human thyroid cell line induces dedifferentiated thyroid carcinogenesis in a mouse xenograft model. Thyroid.

[27] Kurebayashi J, Tanaka K, Otsuki T, Moriya T, Kunisue H, Uno M (2000). All-trans-retinoic acid modulates expression levels of thyroglobulin and cytokines in a new human poorly differentiated papillary thyroid carcinoma cell line, KTC-1. J Clin Endocrinol Metab.

[28] Esmaeili M, Jennek S, Ludwig S, Klitzsch A, Kraft F, Melle C (2016). The tumor suppressor ING1b is a novel corepressor for the androgen receptor and induces cellular senescence in prostate cancer cells. J Mol Cell Biol.

[29] Subramanian A, Tamayo P, Mootha VK, Mukherjee S, Ebert BL, Gillette MA (2005). Gene set enrichment analysis: a knowledge-based approach for interpreting genome-wide expression profiles. Proc Natl Acad Sci U S A.

[30] Camp RL, Dolled-Filhart M, Rimm DL (2004). X-tile: a new bio-informatics tool for biomarker assessment and outcome-based cut-point optimization. Clin Cancer Res.

[31] Vickers AJ, Elkin EB (2006). Decision curve analysis: a novel method for evaluating prediction models. Med Decis Making.

[32] Dom G, Tarabichi M, Unger K, Thomas G, Oczko-Wojciechowska M, Bogdanova T (2012). A gene expression signature distinguishes normal tissues of sporadic and radiation-induced papillary thyroid carcinomas. Br J Cancer.

[33] Wong H, Wong KP, Yau T, Tang V, Leung R, Chiu J (2012). Is there a role for unstimulated thyroglobulin velocity in predicting recurrence in papillary thyroid carcinoma patients with detectable thyroglobulin after radioiodine ablation?. Ann Surg Oncol.

[34] Schmidbauer B, Menhart K, Hellwig D, Grosse J. Differentiated Thyroid Cancer-Treatment: State of the Art.Int J Mol Sci. 2017;18. 10.3390/ijms18061292 PMC5486113 28629126

[35] Cooper DS, Doherty GM, Haugen BR, Kloos RT, Lee SL, Mandel SJ (2009). Revised american thyroid Association Management Guidelines for patients with thyroid nodules and differentiated thyroid Cancer. Thyroid.

[36] Ito Y, Miyauchi A, Oda H (2018). Low-risk papillary microcarcinoma of the thyroid: a review of active surveillance trials. Eur J Surg Oncol.

[37] Saravana-Bawan B, Bajwa A, Paterson J, McMullen T (2020). Active surveillance of low-risk papillary thyroid cancer: a meta-analysis. Surgery.

[38] Cazacu IM, Semaan A, Stephens B, Swartzlander DB, Guerrero PA, Singh BS (2021). Diagnostic value of digital droplet polymerase chain reaction and digital multiplexed detection of single-nucleotide variants in pancreatic cytology specimens collected by EUS-guided FNA. Gastrointest Endosc.

[39] Topilko A, Caillou B (1988). Acetylcholinesterase and butyrylcholinesterase activities in human thyroid cancer cells. Cancer.

[40] Saini S, Sripada L, Tulla K, Qiao G, Kunda N, Maker AV (2019). MADD silencing enhances anti-tumor activity of TRAIL in anaplastic thyroid cancer. Endocr Relat Cancer.

[41] Kulkarni-Gosavi P, Makhoul C, Gleeson PA (2019). Form and function of the golgi apparatus: scaffolds, cytoskeleton and signalling. FEBS Lett.

[42] Parascandolo A, Rappa F, Cappello F, Kim J, Cantu DA, Chen H (2017). Extracellular superoxide dismutase expression in papillary thyroid Cancer mesenchymal Stem/Stromal cells modulates Cancer Cell Growth and Migration. Sci Rep.

[43] Puppin C, Passon N, Frasca F, Vigneri R, Tomay F, Tomaciello S (2011). In thyroid cancer cell lines expression of periostin gene is controlled by p73 and is not related to epigenetic marks of active transcription. Cell Oncol.

[44] Bild A, Febbo PG (2005). Application of a priori established gene sets to discover biologically important differential expression in microarray data. Proc Natl Acad Sci USA.

[45] Balachandran VP, Gonen M, Smith JJ, DeMatteo RP (2015). Nomograms in oncology – more than meets the Eye. Lancet Oncol.

[46] Ho D, Quake SR, McCabe ERB, Chng WJ, Chow EK, Ding X (2020). Enabling Technologies for Personalized and Precision Medicine. Trends Biotechnol.

[47] Yong WP, Rha SY, Tan IB-H, Choo S-P, Syn NL, Koh V (2018). Real-time tumor gene expression profiling to Direct Gastric Cancer Chemotherapy: Proof-of-Concept “3G” trial. Clin Cancer Res.

