# Supplementary material for: Golgi-apparatus genes related signature for predicting the progression-free interval of patients with papillary thyroid carcinoma
Source: BMC Med Genomics. 2023 Mar 27;16:60. doi: 10.1186/s12920-023-01485-z (PMC10041766; doi:10.1186/s12920-023-01485-z)
Supplement: Supplementary file 1 — Supplementary Material 1 [file 12920_2023_1485_MOESM1_ESM.docx]

| Supplementary table 2. 260 DE-GaGs identified in PTC | | | | |
| --- | --- | --- | --- | --- |
| Gene names | logFC | PValue | FDR | Regulated |
| TMEM132A | 1.750697 | 3.70E-20 | 2.06E-15 | Up-Regulated |
| RHBDF1 | 1.09221 | 6.86E-34 | 3.95E-29 | Up-Regulated |
| BAIAP3 | 3.559062 | 6.04E-44 | 3.51E-39 | Up-Regulated |
| SYN1 | 3.105157 | 3.27E-33 | 1.88E-28 | Up-Regulated |
| CAMK1G | 2.08239 | 1.24E-12 | 6.59E-08 | Up-Regulated |
| TENM1 | 4.67923 | 4.49E-57 | 2.62E-52 | Up-Regulated |
| LYPLA2 | 1.029647 | 1.75E-36 | 1.01E-31 | Up-Regulated |
| MAMLD1 | 2.916075 | 1.77E-42 | 1.03E-37 | Up-Regulated |
| CD44 | 1.368109 | 2.85E-42 | 1.66E-37 | Up-Regulated |
| DAPK2 | 1.684223 | 1.13E-22 | 6.34E-18 | Up-Regulated |
| VCAN | 2.058848 | 1.58E-12 | 8.38E-08 | Up-Regulated |
| RAB27B | 3.65389 | 5.25E-20 | 2.92E-15 | Up-Regulated |
| F7 | 1.530203 | 1.54E-09 | 7.80E-05 | Up-Regulated |
| SYT1 | 3.835668 | 3.36E-22 | 1.89E-17 | Up-Regulated |
| FGFR3 | 1.710425 | 7.54E-15 | 4.08E-10 | Up-Regulated |
| ATP11A | 1.597268 | 7.01E-28 | 4.00E-23 | Up-Regulated |
| RAB27A | 1.86793 | 1.05E-43 | 6.09E-39 | Up-Regulated |
| SREBF1 | 1.431653 | 1.08E-41 | 6.27E-37 | Up-Regulated |
| NOTCH3 | 1.262457 | 5.83E-34 | 3.36E-29 | Up-Regulated |
| RBFOX1 | 4.50119 | 3.30E-18 | 1.82E-13 | Up-Regulated |
| FAT2 | 1.978742 | 5.93E-14 | 3.18E-09 | Up-Regulated |
| RPH3A | 2.179303 | 5.46E-10 | 2.79E-05 | Up-Regulated |
| MMP11 | 3.613042 | 1.05E-30 | 6.03E-26 | Up-Regulated |
| KDELR3 | 1.59489 | 3.04E-36 | 1.75E-31 | Up-Regulated |
| PDGFB | 1.021139 | 3.42E-24 | 1.94E-19 | Up-Regulated |
| CABP7 | 1.486135 | 6.84E-07 | 0.032697 | Up-Regulated |
| BIRC7 | 5.66293 | 6.48E-30 | 3.72E-25 | Up-Regulated |
| RNF24 | 1.36583 | 2.03E-59 | 1.19E-54 | Up-Regulated |
| PCSK1N | 5.090613 | 1.97E-35 | 1.14E-30 | Up-Regulated |
| PHEX | 2.219357 | 5.65E-10 | 2.89E-05 | Up-Regulated |
| MSLN | 3.41753 | 1.99E-16 | 1.09E-11 | Up-Regulated |
| STMN2 | 3.848791 | 7.35E-18 | 4.05E-13 | Up-Regulated |
| TNFRSF10A | 1.0081 | 4.70E-18 | 2.59E-13 | Up-Regulated |
| LHB | 1.869058 | 4.47E-12 | 2.35E-07 | Up-Regulated |
| PLD3 | 1.314909 | 2.63E-45 | 1.53E-40 | Up-Regulated |
| CLIP3 | 2.027059 | 7.69E-32 | 4.42E-27 | Up-Regulated |
| APLP1 | 1.204662 | 6.12E-12 | 3.21E-07 | Up-Regulated |
| TGFB1 | 1.543023 | 1.93E-43 | 1.12E-38 | Up-Regulated |
| TMEM59L | 3.535512 | 1.12E-21 | 6.26E-17 | Up-Regulated |
| WNT3 | 1.58431 | 2.23E-30 | 1.28E-25 | Up-Regulated |
| COL1A1 | 2.768862 | 7.07E-17 | 3.88E-12 | Up-Regulated |
| FAM20A | 2.723333 | 3.76E-27 | 2.14E-22 | Up-Regulated |
| RAB34 | 1.212602 | 2.06E-41 | 1.19E-36 | Up-Regulated |
| GALNT7 | 2.326815 | 1.98E-62 | 1.16E-57 | Up-Regulated |
| CTSC | 2.241191 | 2.00E-29 | 1.15E-24 | Up-Regulated |
| B3GAT1 | 2.095571 | 4.30E-36 | 2.48E-31 | Up-Regulated |
| ASIC1 | 1.018379 | 9.78E-10 | 4.98E-05 | Up-Regulated |
| CHPT1 | 1.157553 | 5.27E-27 | 3.00E-22 | Up-Regulated |
| PERP | 1.756636 | 1.92E-32 | 1.10E-27 | Up-Regulated |
| KIF20A | 1.00572 | 1.08E-09 | 5.48E-05 | Up-Regulated |
| ST8SIA4 | 1.561431 | 1.09E-24 | 6.20E-20 | Up-Regulated |
| WNT5A | 1.153014 | 3.86E-16 | 2.10E-11 | Up-Regulated |
| CSPG5 | 1.335922 | 6.14E-20 | 3.42E-15 | Up-Regulated |
| ST3GAL5 | 1.517598 | 1.96E-49 | 1.14E-44 | Up-Regulated |
| PROC | 1.92845 | 2.02E-21 | 1.13E-16 | Up-Regulated |
| SDC1 | 1.178416 | 2.56E-19 | 1.42E-14 | Up-Regulated |
| EPHA4 | 1.370818 | 6.81E-16 | 3.71E-11 | Up-Regulated |
| QSOX1 | 1.728818 | 9.71E-36 | 5.60E-31 | Up-Regulated |
| ARHGEF2 | 1.023695 | 1.92E-43 | 1.12E-38 | Up-Regulated |
| ST6GALNAC5 | 5.446822 | 4.27E-31 | 2.45E-26 | Up-Regulated |
| GBP3 | 1.169826 | 3.67E-12 | 1.93E-07 | Up-Regulated |
| MUC5B | 1.787307 | 1.93E-07 | 0.009391 | Up-Regulated |
| B4GALT6 | 1.501669 | 6.85E-24 | 3.87E-19 | Up-Regulated |
| SPP1 | 1.66152 | 2.43E-09 | 0.000123 | Up-Regulated |
| TGFBI | 1.958664 | 4.87E-24 | 2.75E-19 | Up-Regulated |
| CLU | 1.633687 | 8.43E-17 | 4.62E-12 | Up-Regulated |
| NPY | 4.019782 | 6.51E-09 | 0.000327 | Up-Regulated |
| KCNJ2 | 3.925339 | 6.98E-46 | 4.06E-41 | Up-Regulated |
| SLPI | 3.807219 | 3.20E-23 | 1.80E-18 | Up-Regulated |
| SDC4 | 2.482658 | 6.24E-43 | 3.62E-38 | Up-Regulated |
| PMEPA1 | 1.360928 | 2.89E-35 | 1.67E-30 | Up-Regulated |
| MYRF | 2.657504 | 3.47E-16 | 1.89E-11 | Up-Regulated |
| PLA2G5 | 1.64929 | 3.99E-09 | 0.000201 | Up-Regulated |
| PKMYT1 | 1.070089 | 1.27E-12 | 6.72E-08 | Up-Regulated |
| HIP1 | 1.308522 | 7.35E-40 | 4.26E-35 | Up-Regulated |
| STEAP4 | 1.102476 | 3.99E-14 | 2.14E-09 | Up-Regulated |
| MGAT3 | 3.51262 | 5.24E-33 | 3.01E-28 | Up-Regulated |
| VGF | 4.373591 | 2.37E-13 | 1.27E-08 | Up-Regulated |
| MTUS1 | 1.228618 | 8.61E-32 | 4.95E-27 | Up-Regulated |
| CDH15 | 3.240093 | 2.21E-27 | 1.26E-22 | Up-Regulated |
| APOE | 2.240586 | 1.14E-15 | 6.18E-11 | Up-Regulated |
| GDF15 | 4.267353 | 5.70E-47 | 3.32E-42 | Up-Regulated |
| TRIM22 | 1.083263 | 1.06E-13 | 5.70E-09 | Up-Regulated |
| SNAP25 | 1.737309 | 4.90E-13 | 2.61E-08 | Up-Regulated |
| BCAN | 3.501323 | 7.56E-12 | 3.97E-07 | Up-Regulated |
| ATP8A2 | 1.150314 | 3.33E-09 | 0.000168 | Up-Regulated |
| POSTN | 3.038131 | 1.52E-20 | 8.49E-16 | Up-Regulated |
| EHF | 1.265977 | 4.31E-07 | 0.020722 | Up-Regulated |
| GALNT5 | 3.519344 | 3.73E-24 | 2.11E-19 | Up-Regulated |
| CDK5RAP2 | 1.071496 | 3.84E-14 | 2.06E-09 | Up-Regulated |
| TLR2 | 1.159594 | 4.04E-14 | 2.17E-09 | Up-Regulated |
| B4GALNT3 | 1.850865 | 9.74E-31 | 5.59E-26 | Up-Regulated |
| RAB15 | 1.273265 | 7.77E-32 | 4.46E-27 | Up-Regulated |
| ST8SIA2 | 1.82575 | 2.30E-11 | 1.20E-06 | Up-Regulated |
| TMC6 | 2.17686 | 1.77E-42 | 1.03E-37 | Up-Regulated |
| FCGR2A | 1.142699 | 1.97E-11 | 1.03E-06 | Up-Regulated |
| XPR1 | 1.705844 | 6.61E-49 | 3.84E-44 | Up-Regulated |
| MALL | 1.111197 | 1.41E-09 | 7.14E-05 | Up-Regulated |
| GALNT13 | 2.576096 | 7.91E-12 | 4.15E-07 | Up-Regulated |
| ABCA12 | 1.701686 | 4.04E-16 | 2.21E-11 | Up-Regulated |
| IL17RD | 2.20547 | 1.17E-33 | 6.76E-29 | Up-Regulated |
| MUC4 | 1.583726 | 1.13E-08 | 0.000567 | Up-Regulated |
| AHSG | 3.719947 | 1.54E-14 | 8.32E-10 | Up-Regulated |
| OCIAD2 | 1.011594 | 1.87E-29 | 1.07E-24 | Up-Regulated |
| RNF175 | 1.396193 | 2.63E-12 | 1.39E-07 | Up-Regulated |
| PAM | 1.040615 | 6.53E-27 | 3.72E-22 | Up-Regulated |
| CXCL14 | 4.006001 | 2.51E-17 | 1.38E-12 | Up-Regulated |
| CLVS2 | 2.919092 | 2.01E-07 | 0.00975 | Up-Regulated |
| IGFBP1 | 1.736341 | 1.51E-07 | 0.007368 | Up-Regulated |
| RNF144A | 1.104577 | 4.68E-18 | 2.58E-13 | Up-Regulated |
| WNT3A | 2.419505 | 2.10E-08 | 0.001042 | Up-Regulated |
| WNT7A | 2.968811 | 3.77E-12 | 1.98E-07 | Up-Regulated |
| PI4K2A | 1.130904 | 4.05E-29 | 2.32E-24 | Up-Regulated |
| GOLGA7B | 1.907946 | 4.59E-17 | 2.52E-12 | Up-Regulated |
| MMP16 | 2.93903 | 2.27E-28 | 1.30E-23 | Up-Regulated |
| B3GNT7 | 2.63809 | 4.93E-50 | 2.87E-45 | Up-Regulated |
| MMP14 | 1.112013 | 1.96E-16 | 1.07E-11 | Up-Regulated |
| ACAN | 2.580193 | 2.72E-24 | 1.54E-19 | Up-Regulated |
| EXTL1 | 1.244013 | 1.76E-17 | 9.70E-13 | Up-Regulated |
| AGRP | 1.556748 | 1.95E-08 | 0.000971 | Up-Regulated |
| PDE9A | 1.779425 | 3.72E-43 | 2.16E-38 | Up-Regulated |
| S100A1 | 1.907798 | 1.51E-29 | 8.66E-25 | Up-Regulated |
| COL26A1 | 1.76577 | 2.13E-13 | 1.14E-08 | Up-Regulated |
| MGAT4B | 1.265101 | 2.07E-42 | 1.20E-37 | Up-Regulated |
| HS3ST6 | 2.214141 | 2.64E-11 | 1.37E-06 | Up-Regulated |
| SDC3 | 1.469192 | 4.08E-35 | 2.35E-30 | Up-Regulated |
| SPRR3 | 5.004484 | 1.47E-13 | 7.88E-09 | Up-Regulated |
| ELF3 | 2.232296 | 7.96E-23 | 4.48E-18 | Up-Regulated |
| TMEM79 | 2.158963 | 8.51E-42 | 4.93E-37 | Up-Regulated |
| SPRY1 | 1.237077 | 4.54E-22 | 2.55E-17 | Up-Regulated |
| RHOBTB3 | 1.257187 | 1.64E-30 | 9.41E-26 | Up-Regulated |
| SHH | 3.258091 | 1.95E-11 | 1.02E-06 | Up-Regulated |
| DEFB1 | 1.970631 | 6.82E-08 | 0.003354 | Up-Regulated |
| NOS3 | 1.055792 | 4.40E-23 | 2.48E-18 | Up-Regulated |
| RNF183 | 3.741789 | 4.00E-30 | 2.29E-25 | Up-Regulated |
| TMEM130 | 3.687132 | 1.66E-27 | 9.44E-23 | Up-Regulated |
| MS4A6E | 2.219756 | 4.97E-09 | 0.00025 | Up-Regulated |
| KLK11 | 7.064528 | 5.48E-28 | 3.13E-23 | Up-Regulated |
| IGFBP6 | 2.71069 | 1.41E-22 | 7.94E-18 | Up-Regulated |
| BMP1 | 1.591066 | 2.06E-68 | 1.20E-63 | Up-Regulated |
| GDNF | 3.496686 | 3.05E-15 | 1.65E-10 | Up-Regulated |
| PCSK9 | 2.550175 | 1.91E-11 | 1.00E-06 | Up-Regulated |
| RAB3B | 1.560301 | 3.18E-09 | 0.000161 | Up-Regulated |
| KCNS3 | 1.670685 | 1.39E-28 | 7.95E-24 | Up-Regulated |
| HS6ST2 | 3.66169 | 3.29E-18 | 1.82E-13 | Up-Regulated |
| FUT3 | 5.413666 | 8.63E-34 | 4.97E-29 | Up-Regulated |
| CHST11 | 1.065556 | 2.22E-19 | 1.23E-14 | Up-Regulated |
| FUT9 | 5.769345 | 9.71E-19 | 5.38E-14 | Up-Regulated |
| CSPG4 | 1.655361 | 3.09E-28 | 1.76E-23 | Up-Regulated |
| TRAPPC3L | 1.790812 | 3.55E-08 | 0.001757 | Up-Regulated |
| PLK3 | 1.156684 | 1.38E-19 | 7.68E-15 | Up-Regulated |
| GALNTL6 | 2.820099 | 1.87E-30 | 1.07E-25 | Up-Regulated |
| GOLT1A | 4.00799 | 3.13E-73 | 1.83E-68 | Up-Regulated |
| FUT1 | 1.03455 | 4.41E-30 | 2.53E-25 | Up-Regulated |
| CHST2 | 2.274852 | 4.09E-39 | 2.37E-34 | Up-Regulated |
| GOLGA8A | 1.140723 | 1.29E-09 | 6.55E-05 | Up-Regulated |
| CREG2 | 2.52473 | 2.77E-28 | 1.58E-23 | Up-Regulated |
| FUT2 | 1.524302 | 5.32E-19 | 2.95E-14 | Up-Regulated |
| GCNT4 | 1.2122 | 4.06E-11 | 2.11E-06 | Up-Regulated |
| MUC20 | 1.309292 | 7.57E-15 | 4.09E-10 | Up-Regulated |
| ACER2 | 1.345456 | 2.07E-14 | 1.11E-09 | Up-Regulated |
| B3GNT8 | 2.266812 | 2.16E-43 | 1.26E-38 | Up-Regulated |
| FAM20C | 1.18257 | 6.61E-19 | 3.66E-14 | Up-Regulated |
| B3GNT3 | 5.973488 | 6.09E-38 | 3.52E-33 | Up-Regulated |
| CHST13 | 1.292743 | 8.00E-10 | 4.08E-05 | Up-Regulated |
| F2R | 1.496941 | 2.40E-32 | 1.38E-27 | Up-Regulated |
| ATG9B | 1.114478 | 2.73E-09 | 0.000138 | Up-Regulated |
| BGN | 1.011869 | 1.28E-10 | 6.59E-06 | Up-Regulated |
| HS3ST4 | 2.041165 | 1.53E-07 | 0.007448 | Up-Regulated |
| CHST6 | 2.746802 | 1.13E-23 | 6.39E-19 | Up-Regulated |
| SYNDIG1L | 3.234697 | 3.53E-37 | 2.04E-32 | Up-Regulated |
| PROS1 | 3.600447 | 9.75E-53 | 5.68E-48 | Up-Regulated |
| MANEAL | 1.852265 | 4.09E-50 | 2.38E-45 | Up-Regulated |
| MUC1 | 2.522309 | 3.13E-18 | 1.73E-13 | Up-Regulated |
| SLC18A3 | 7.327197 | 8.14E-20 | 4.53E-15 | Up-Regulated |
| S100A3 | 1.080979 | 3.80E-10 | 1.95E-05 | Up-Regulated |
| WNT7B | 1.063891 | 1.34E-10 | 6.92E-06 | Up-Regulated |
| AGRN | 1.207838 | 1.45E-50 | 8.42E-46 | Up-Regulated |
| SLC24A5 | 2.414813 | 3.28E-07 | 0.01582 | Up-Regulated |
| ASAH2 | 1.450689 | 1.33E-07 | 0.006476 | Up-Regulated |
| LRRK2 | 4.057065 | 1.06E-50 | 6.18E-46 | Up-Regulated |
| SFTA2 | 2.073196 | 1.07E-13 | 5.75E-09 | Up-Regulated |
| CD55 | 2.590704 | 2.27E-25 | 1.29E-20 | Up-Regulated |
| FLNA | 1.02027 | 1.26E-17 | 6.95E-13 | Up-Regulated |
| SERPINA1 | 4.696984 | 3.78E-47 | 2.20E-42 | Up-Regulated |
| PDGFA | 1.368577 | 1.02E-45 | 5.93E-41 | Up-Regulated |
| B3GNT6 | 3.164987 | 1.03E-12 | 5.47E-08 | Up-Regulated |
| CAPN8 | 2.75013 | 2.52E-19 | 1.40E-14 | Up-Regulated |
| MUC21 | 6.281059 | 2.55E-30 | 1.46E-25 | Up-Regulated |
| HLA-G | 2.347544 | 7.83E-14 | 4.20E-09 | Up-Regulated |
| MUC19 | 1.400593 | 3.44E-07 | 0.016576 | Up-Regulated |
| GPC2 | 1.126617 | 9.23E-10 | 4.71E-05 | Up-Regulated |
| EMP2 | 1.149301 | 2.25E-22 | 1.26E-17 | Up-Regulated |
| MUC5AC | 2.133448 | 4.44E-07 | 0.021362 | Up-Regulated |
| HSPB6 | -1.52439 | 6.47E-23 | 3.64E-18 | Down-Regulated |
| CYTH3 | -1.00956 | 1.87E-45 | 1.09E-40 | Down-Regulated |
| DCN | -1.83553 | 9.67E-19 | 5.36E-14 | Down-Regulated |
| MYOC | -3.36299 | 1.62E-32 | 9.34E-28 | Down-Regulated |
| ATP2C2 | -2.47366 | 5.22E-55 | 3.04E-50 | Down-Regulated |
| FGFR2 | -1.06185 | 1.57E-29 | 8.97E-25 | Down-Regulated |
| ABCB11 | -1.8199 | 7.67E-10 | 3.92E-05 | Down-Regulated |
| RAP1GAP | -1.2656 | 3.82E-17 | 2.10E-12 | Down-Regulated |
| LRP2 | -1.33277 | 1.99E-16 | 1.09E-11 | Down-Regulated |
| PPP1R15A | -1.04838 | 1.89E-22 | 1.06E-17 | Down-Regulated |
| OGN | -2.19728 | 4.10E-19 | 2.28E-14 | Down-Regulated |
| SOD3 | -1.38359 | 1.30E-14 | 7.01E-10 | Down-Regulated |
| CRYAB | -1.21726 | 3.84E-18 | 2.12E-13 | Down-Regulated |
| GBP1 | -1.54845 | 2.18E-29 | 1.25E-24 | Down-Regulated |
| MPL | -1.11612 | 3.34E-32 | 1.92E-27 | Down-Regulated |
| OLFM3 | -1.65697 | 8.61E-10 | 4.39E-05 | Down-Regulated |
| FGF23 | -1.68172 | 2.58E-08 | 0.00128 | Down-Regulated |
| CCDC170 | -1.05568 | 2.06E-25 | 1.17E-20 | Down-Regulated |
| GLT8D2 | -1.82222 | 2.56E-32 | 1.47E-27 | Down-Regulated |
| FMOD | -1.37546 | 7.87E-29 | 4.50E-24 | Down-Regulated |
| F10 | -2.18323 | 2.42E-58 | 1.41E-53 | Down-Regulated |
| PROZ | -1.05325 | 1.58E-12 | 8.36E-08 | Down-Regulated |
| OMD | -1.68008 | 2.77E-11 | 1.44E-06 | Down-Regulated |
| SYNE1 | -1.61787 | 1.94E-41 | 1.12E-36 | Down-Regulated |
| SYT4 | -2.17983 | 2.83E-23 | 1.60E-18 | Down-Regulated |
| PDGFRA | -1.07946 | 1.60E-08 | 0.000799 | Down-Regulated |
| STX11 | -1.07258 | 3.56E-17 | 1.95E-12 | Down-Regulated |
| FHDC1 | -1.34362 | 8.43E-40 | 4.88E-35 | Down-Regulated |
| MTTP | -1.15257 | 6.80E-10 | 3.48E-05 | Down-Regulated |
| FGF7 | -1.13454 | 9.58E-07 | 0.045576 | Down-Regulated |
| PCSK6 | -1.04813 | 5.88E-13 | 3.12E-08 | Down-Regulated |
| GPC3 | -3.06637 | 5.41E-67 | 3.16E-62 | Down-Regulated |
| CSGALNACT1 | -1.79399 | 3.67E-55 | 2.14E-50 | Down-Regulated |
| NCAM1 | -1.51809 | 5.87E-15 | 3.18E-10 | Down-Regulated |
| GFRA1 | -2.08893 | 3.18E-23 | 1.79E-18 | Down-Regulated |
| CHST9 | -1.68279 | 1.80E-13 | 9.60E-09 | Down-Regulated |
| ABCA6 | -1.55964 | 3.70E-27 | 2.11E-22 | Down-Regulated |
| SH3RF1 | -1.03149 | 9.32E-41 | 5.40E-36 | Down-Regulated |
| STEAP2 | -1.08811 | 1.09E-12 | 5.75E-08 | Down-Regulated |
| CLSTN2 | -1.41553 | 6.30E-14 | 3.38E-09 | Down-Regulated |
| B3GALT2 | -1.62676 | 4.24E-11 | 2.20E-06 | Down-Regulated |
| B4GALNT2 | -1.82068 | 3.12E-15 | 1.69E-10 | Down-Regulated |
| SOST | -3.11222 | 2.63E-25 | 1.49E-20 | Down-Regulated |
| BDKRB2 | -1.01595 | 1.88E-12 | 9.95E-08 | Down-Regulated |
| NLGN1 | -2.08934 | 3.42E-33 | 1.97E-28 | Down-Regulated |
| TPPP | -2.09192 | 2.92E-67 | 1.70E-62 | Down-Regulated |
| DSEL | -1.00117 | 8.29E-28 | 4.73E-23 | Down-Regulated |
| PCSK1 | -1.13829 | 6.02E-13 | 3.19E-08 | Down-Regulated |
| FIBIN | -1.30801 | 6.61E-10 | 3.38E-05 | Down-Regulated |
| CLVS1 | -1.38513 | 4.90E-21 | 2.74E-16 | Down-Regulated |
| ST8SIA3 | -2.12615 | 6.90E-11 | 3.57E-06 | Down-Regulated |
| GOLGA8Q | -1.50886 | 9.46E-11 | 4.89E-06 | Down-Regulated |
| GALNT9 | -1.19314 | 2.59E-10 | 1.33E-05 | Down-Regulated |
| B3GALT5 | -1.38217 | 2.55E-10 | 1.31E-05 | Down-Regulated |
| COLEC10 | -1.63384 | 1.59E-25 | 9.03E-21 | Down-Regulated |
| WWOX | -1.01516 | 2.37E-39 | 1.37E-34 | Down-Regulated |
| GOLGA8R | -1.32149 | 1.24E-15 | 6.74E-11 | Down-Regulated |
| JAKMIP3 | -1.05816 | 5.65E-12 | 2.97E-07 | Down-Regulated |
| GOLGA8M | -1.18282 | 1.27E-08 | 0.000635 | Down-Regulated |
| ZDHHC11 | -1.0949 | 2.71E-18 | 1.50E-13 | Down-Regulated |
| CD247 | -1.06383 | 7.58E-09 | 0.00038 | Down-Regulated |
| ZDHHC11B | -1.43728 | 8.12E-30 | 4.65E-25 | Down-Regulated |
| GOLGA8O | -1.56517 | 1.56E-13 | 8.35E-09 | Down-Regulated |
| GOLGA8K | -1.21618 | 1.50E-14 | 8.11E-10 | Down-Regulated |
| GOLGA8T | -1.19424 | 3.34E-12 | 1.76E-07 | Down-Regulated |
| GOLGA8IP | -1.18519 | 2.83E-10 | 1.45E-05 | Down-Regulated |
