# Supplementary material for: Golgi-apparatus genes related signature for predicting the progression-free interval of patients with papillary thyroid carcinoma
Source: BMC Med Genomics. 2023 Mar 27;16:60. doi: 10.1186/s12920-023-01485-z (PMC10041766; doi:10.1186/s12920-023-01485-z)
Supplement: Supplementary file 2 — Supplementary Material 2 [file 12920_2023_1485_MOESM2_ESM.docx]

| Supplementary table 1. The experimentally curated GaGs from MsigDB |
| --- |
| Genes |
| A3GALT2, A4GALT, A4GNT, AATF, ABCA1, ABCA12, ABCA5, ABCA6, ABCA7, ABCB11, ABCB6, ABCC5, ABCG1, ABO, ACAN, ACBD3, ACER2, ACER3, ACHE, ACO1, ACSL3, ACTL7A, ADAM10, ADAM17, ADAM19, ADCY3, ADRB2, AFTPH, AGBL4, AGPAT3, AGRN, AGRP, AGTRAP, AHSG, AIMP1, AJUBA, AKAP8, AKAP9, AKR7A2, ALB, ALKBH5, ALX1, AMELX, AMFR, ANGEL1, ANGPTL3, ANK3, AOC3, AP1AR, AP1B1, AP1G1, AP1G2, AP1M1, AP1M2, AP1S1, AP1S2, AP1S3, AP2A1, AP3B1, AP3B2, AP3D1, AP3M1, AP3M2, AP3S1, AP3S2, AP4B1, AP4E1, AP4M1, AP4S1, APBA1, APBB2, APC, APC2, APH1A, APLP1, APOA5, APOE, APOO, APP, AQP2, ARAP1, ARCN1, ARF1, ARF3, ARF4, ARF5, ARF6, ARFGAP1, ARFGAP2, ARFGAP3, ARFGEF1, ARFGEF2, ARFIP1, ARFIP2, ARFRP1, ARHGAP21, ARHGAP32, ARHGAP4, ARHGEF2, ARL1, ARL17B, ARL2, ARL3, ARL5A, ARL5B, ARL5C, ARMH3, ARRB1, ARSB, ARSL, ARV1, ASAH2, ASAP2, ASH1L, ASIC1, ATAT1, ATF6, ATF6B, ATG9A, ATG9B, ATL1, ATP11A, ATP11B, ATP11C, ATP1A1, ATP1A3, ATP2C1, ATP2C2, ATP6V0A1, ATP7A, ATP7B, ATP8A1, ATP8A2, ATP8B1, ATP8B2, ATP8B3, ATP8B4, ATP9A, ATP9B, ATR, ATXN2, AVPR1B, AVPR2, AZIN2, B2M, B3GALNT1, B3GALNT2, B3GALT1, B3GALT2, B3GALT4, B3GALT5, B3GALT6, B3GALT9, B3GAT1, B3GAT2, B3GAT3, B3GNT2, B3GNT3, B3GNT4, B3GNT5, B3GNT6, B3GNT7, B3GNT8, B3GNT9, B4GALNT1, B4GALNT2, B4GALNT3, B4GALNT4, B4GALT1, B4GALT2, B4GALT3, B4GALT4, B4GALT5, B4GALT6, B4GALT7, B4GAT1, BACE1, BACE2, BAIAP2, BAIAP3, BARX2, BCAN, BCAP31, BCL6, BCL9, BDKRB2, BECN1, BEND5, BET1, BET1L, BGLAP, BGN, BICD1, BICD2, BIRC6, BIRC7, BLZF1, BMP1, BOK, BPNT2, BSG, BST2, C11orf24, C17orf75, C1GALT1, C1GALT1C1, C1orf43, C6orf89, CA4, CABP1, CABP2, CABP7, CALN1, CALR, CALU, CAMK1G, CAMKMT, CAMSAP2, CAND1, CANT1, CAPN2, CAPN8, CASD1, CASQ1, CAT, CAV1, CAV2, CAV3, CBFA2T3, CBL, CBLN3, CBY1, CCDC170, CCDC186, CCDC88A, CCDC88B, CCDC91, CCN2, CD14, CD1B, CD1E, CD247, CD33, CD36, CD44, CD55, CD59, CD74, CDC42, CDH1, CDH15, CDIPT, CDK13, CDK5RAP2, CEP57, CEP83, CEP85, CEPT1, CERKL, CERT1, CGA, CHAC1, CHEK2, CHIC2, CHID1, CHP1, CHPF, CHPF2, CHPT1, CHST1, CHST10, CHST11, CHST12, CHST13, CHST14, CHST15, CHST2, CHST3, CHST4, CHST5, CHST6, CHST7, CHST8, CHST9, CHSY1, CHSY3, CIB1, CIT, CLASP1, CLASP2, CLBA1, CLCC1, CLCN3, CLCN4, CLCN5, CLEC16A, CLEC18A, CLEC18B, CLEC18C, CLIC5, CLINT1, CLIP3, CLN3, CLN5, CLSPN, CLSTN1, CLSTN2, CLSTN3, CLTA, CLTB, CLTC, CLTCL1, CLU, CLVS1, CLVS2, CNIH1, CNST, CNTNAP2, COG1, COG2, COG3, COG4, COG5, COG6, COG7, COG8, COL1A1, COL26A1, COLEC10, COMMD9, COPA, COPB1, COPB2, COPE, COPG1, COPG2, COPZ1, COPZ2, COQ6, CORO7, CPE, CPQ, CPTP, CRACR2A, CREB3, CREB3L3, CREB3L4, CREG2, CRELD2, CRHBP, CRYAB, CRYZL2P-SEC16B, CSDE1, CSGALNACT1, CSGALNACT2, CSNK1D, CSPG4, CSPG5, CST3, CST7, CTLA4, CTSC, CTSL, CTTN, CUBN, CUL3, CUL7, CUX1, CXCL14, CYBA, CYBB, CYP2E1, CYTH1, CYTH2, CYTH3, CYTH4, DAG1, DAOA, DAPK2, DBI, DBNL, DCLRE1C, DCN, DDHD2, DDX31, DDX54, DEFA1, DEFA1B, DEFA3, DEFA4, DEFA5, DEFA6, DEFB1, DEFB103A, DEFB103B, DEFB4A, DENND4B, DENND4C, DENND5A, DHCR24, DIPK2A, DLG1, DNAAF6, DNAJC28, DNAJC5, DNM1L, DNM2, DNM3, DNMBP, DOCK4, DOP1A, DOP1B, DPP7, DPY30, DRAM2, DRD2, DSE, DSEL, DUSP10, DUSP26, DUX4, DYM, DYNAP, DYNC2H1, DYNC2LI1, DYNLT1, EBAG9, ECE2, ECPAS, EEF1AKMT4-ECE2, EGFR, EHF, EI24, EIPR1, ELAPOR1, ELF3, EMID1, EMP2, ENPP7, ENTPD4, ENTPD6, ENTPD7, EPHA4, EPHB2, ERC1, ERGIC1, ERGIC2, ERGIC3, ERMAP, ESCO2, ESR1, EXOC3, EXT1, EXT2, EXTL1, EXTL3, F10, F2, F2R, F2RL1, F5, F7, F8, F9, FAIM2, FAM114A1, FAM174B, FAM20A, FAM20B, FAM20C, FAM234B, FAM241A, FAM3C, FAM91A1, FASN, FAT2, FBXW7, FBXW8, FCGR2A, FES, FEZ1, FGD1, FGD2, FGD3, FGD4, FGD5, FGD6, FGF23, FGF7, FGFR2, FGFR3, FGFR4, FGFRL1, FHDC1, FIBIN, FIG4, FKRP, FKTN, FLNA, FMNL3, FMOD, FNDC3A, FOLR1, FTCD, FURIN, FUT1, FUT10, FUT11, FUT2, FUT3, FUT4, FUT5, FUT6, FUT7, FUT8, FUT9, FYCO1, FZD5, FZD8, FZD9, G2E3, GABARAP, GABARAPL1, GABARAPL2, GAD2, GAK, GAL, GAL3ST1, GAL3ST2, GAL3ST3, GAL3ST4, GALNT1, GALNT10, GALNT11, GALNT12, GALNT13, GALNT14, GALNT15, GALNT16, GALNT17, GALNT18, GALNT2, GALNT3, GALNT4, GALNT5, GALNT6, GALNT7, GALNT8, GALNT9, GALNTL5, GALNTL6, GALT, GANAB, GAPT, GAS6, GAS8, GASK1A, GASK1B, GBA, GBA2, GBF1, GBGT1, GBP1, GBP2, GBP3, GBP4, GBP5, GCC1, GCC2, GCNT1, GCNT2, GCNT3, GCNT4, GCNT7, GDF15, GDI1, GDI2, GDNF, GFRA1, GFY, GGA1, GGA2, GGA3, GGNBP1, GGTA1, GIGYF2, GIMAP1, GIMAP7, GIMAP8, GJA1, GKAP1, GKN1, GLA, GLB1, GLCE, GLG1, GLIPR2, GLT6D1, GLT8D1, GLT8D2, GLYCTK, GNAI3, GNAQ, GNPNAT1, GNPTAB, GNPTG, GOLGA1, GOLGA2, GOLGA2P5, GOLGA3, GOLGA4, GOLGA5, GOLGA6A, GOLGA6B, GOLGA6C, GOLGA6D, GOLGA6L7, GOLGA7, GOLGA7B, GOLGA8A, GOLGA8B, GOLGA8CP, GOLGA8DP, GOLGA8G, GOLGA8H, GOLGA8IP, GOLGA8J, GOLGA8K, GOLGA8M, GOLGA8N, GOLGA8O, GOLGA8Q, GOLGA8R, GOLGA8S, GOLGA8T, GOLGB1, GOLIM4, GOLM1, GOLM2, GOLPH3, GOLPH3L, GOLT1A, GOLT1B, GOPC, GORAB, GORASP1, GORASP2, GOSR1, GOSR2, GPC1, GPC2, GPC3, GPC4, GPC5, GPC6, GPER1, GPR107, GPR108, GPR143, GPR89A, GPR89B, GPSM1, GRB2, GRINA, GRM6, GRN, GSAP, H1-0, HABP4, HACE1, HCK, HDAC3, HDAC5, HEPACAM2, HERC1, HHAT, HID1, HIP1, HLA-A, HLA-B, HLA-C, HLA-DPA1, HLA-DPB1, HLA-DQA1, HLA-DQA2, HLA-DQB1, HLA-DQB2, HLA-DRA, HLA-DRB1, HLA-DRB3, HLA-DRB4, HLA-DRB5, HLA-E, HLA-F, HLA-G, HLA-H, HOOK2, HOOK3, HPD, HRAS, HS2ST1, HS3ST1, HS3ST2, HS3ST3A1, HS3ST3B1, HS3ST4, HS3ST5, HS3ST6, HS6ST1, HS6ST2, HSPB6, HSPG2, HTR5A, HTR7, HTT, HUWE1, HYAL2, ICA1, ICA1L, ID1, IER3IP1, IFNGR2, IFT20, IFT27, IFT57, IGF2R, IGFBP1, IGFBP6, IL15, IL15RA, IL17RD, INPP5B, INPP5E, INPP5K, INPPL1, INS, IRGM, ITGA5, ITM2A, ITM2B, ITM2C, JAKMIP2, JAKMIP3, JAM3, KBTBD8, KCNA5, KCNE2, KCNIP3, KCNJ2, KCNJ6, KCNS3, KDELR1, KDELR2, KDELR3, KDR, KERA, KIAA0319L, KIF13A, KIF1C, KIF20A, KIFAP3, KIFC3, KLF5, KLHL20, KLK11, KPNA2, KRAS, LALBA, LAP3, LAPTM4A, LARGE1, LARGE2, LAT, LAX1, LDLR, LDLRAD4, LEPROT, LFNG, LGR5, LGR6, LHB, LIMK2, LINC00869, LIPG, LITAF, LLGL1, LMAN1, LMAN1L, LMAN2, LMAN2L, LMTK2, LMTK3, LPCAT1, LPCAT2, LRBA, LRP1, LRP2, LRP6, LRPAP1, LRRK2, LST1, LTBR, LUM, LYN, LYPLA2, LYSMD3, LZTR1, M6PR, MACF1, MALL, MALRD1, MAMDC4, MAMLD1, MAN1A1, MAN1A2, MAN1B1, MAN1C1, MAN2A1, MAN2A2, MANEA, MANEAL, MAP2K1, MAP2K2, MAP4K2, MAP6, MAP6D1, MAPK1, MAPK15, MAPK3, MAPK8IP3, MAPKAP1, MAPRE1, MARCHF1, MARCHF2, MARCHF4, MARCHF9, MARF1, MBTPS1, MBTPS2, MCFD2, MCOLN1, MDGA1, MFNG, MGAT1, MGAT2, MGAT3, MGAT4A, MGAT4B, MGAT4C, MGAT4D, MGAT5, MGAT5B, MICALL1, MIF4GD, MINK1, MLANA, MMD, MMD2, MME, MMGT1, MMP11, MMP14, MMP16, MMP24, MOB4, MOSMO, MOSPD1, MPHOSPH9, MPIG6B, MPL, MPLKIP, MPPE1, MR1, MS4A4A, MS4A4E, MS4A6A, MS4A6E, MS4A7, MSH6, MSLN, MTOR, MTTP, MTUS1, MUC1, MUC12, MUC13, MUC15, MUC16, MUC17, MUC19, MUC2, MUC20, MUC21, MUC3A, MUC4, MUC5AC, MUC5B, MUC6, MUC7, MUCL1, MVB12A, MYDGF, MYMK, MYMX, MYO18A, MYO1B, MYO6, MYOC, MYRF, NAA11, NAA25, NAA60, NAGPA, NAPEPLD, NBEA, NCALD, NCAM1, NCAN, NCF1, NCS1, NCSTN, NDFIP1, NDFIP2, NDRG2, NDST1, NDST2, NDST3, NDST4, NECAB3, NEDD4, NEDD4L, NEDD9, NEO1, NFE2L2, NGF, NHS, NLGN1, NLRP2, NLRP3, NLRP5, NMNAT2, NMT2, NOD2, NOS3, NOSIP, NOTCH1, NOTCH2, NOTCH3, NOTCH4, NPC1, NPY, NRAS, NSF, NSFL1C, NSG1, NSG2, NTN3, NTSR1, NUCB1, NUCB2, NUDC, NUMA1, OBSL1, OCIAD1, OCIAD2, OCRL, OGN, OLFM3, OMD, OPALIN, OPRM1, OPTN, OSBP, OSBPL11, OSBPL9, OTOF, P2RX3, P3H2, PACS1, PAK4, PALS1, PAM, PAQR3, PAQR8, PARM1, PARP10, PCGF5, PCSK1, PCSK1N, PCSK4, PCSK5, PCSK6, PCSK7, PCSK9, PDCD10, PDE2A, PDE3B, PDE4DIP, PDE9A, PDGFA, PDGFB, PDGFC, PDGFD, PDGFRA, PDGFRB, PDXDC1, PERP, PEX5, PGAP2, PGAP3, PGAP4, PHAF1, PHETA1, PHETA2, PHEX, PHF7, PHTF1, PI4K2A, PI4K2B, PI4KB, PICALM, PICK1, PIDD1, PIFO, PIK3C2A, PIK3R1, PIKFYVE, PITPNB, PITPNM1, PJA2, PKD1, PKD2, PKDCC, PKHD1, PKMYT1, PKN3, PLA2G4A, PLA2G5, PLAGL1, PLCB2, PLCE1, PLD1, PLD3, PLD4, PLD6, PLEKHA3, PLEKHA8, PLEKHJ1, PLEKHM3, PLIN3, PLK3, PLOD3, PLPP3, PLSCR1, PMEL, PMEPA1, PMF1, PNRC2, PODXL2, POFUT2, POLQ, POMGNT1, POSTN, PPHLN1, PPIL2, PPP1R15A, PPP2R3C, PPP2R5C, PPT1, PRAME, PRCD, PRDM2, PRELP, PREPL, PRKAA2, PRKCE, PRKCI, PRKD1, PRKD2, PRKG1, PRKN, PRMT5, PRNP, PROC, PROS1, PROZ, PRRC1, PSEN1, PSEN2, PSENEN, PSKH1, PSMG1, PTCH1, PTGDS, PTGES2, PTGFRN, PTGS1, PTHLH, PTPRN, PWP1, PXYLP1, PYCARD, QPCTL, QSOX1, QSOX2, RAB10, RAB11A, RAB11FIP5, RAB12, RAB13, RAB14, RAB15, RAB18, RAB1A, RAB1B, RAB20, RAB21, RAB26, RAB27A, RAB27B, RAB29, RAB2A, RAB2B, RAB30, RAB31, RAB32, RAB33A, RAB33B, RAB34, RAB36, RAB37, RAB38, RAB39A, RAB39B, RAB3B, RAB3GAP1, RAB3IP, RAB40A, RAB40C, RAB41, RAB43, RAB6A, RAB6B, RAB6C, RAB6D, RAB7A, RAB7B, RAB8A, RAB9A, RABAC1, RABEPK, RABGAP1L, RAC1, RAF1, RAP1GAP, RAPSN, RASGRP1, RASIP1, RASSF2, RBFOX1, RELCH, RER1, RESP18, RETREG1, RFFL, RFNG, RGP1, RGS19, RGS20, RHBDD2, RHBDF1, RHBDF2, RHEB, RHO, RHOBTB3, RHOD, RHOU, RIC1, RIC3, RIPOR1, RMDN2, RND3, RNF121, RNF122, RNF125, RNF128, RNF13, RNF133, RNF144A, RNF148, RNF175, RNF183, RNF215, RNF24, ROCK1, RP2, RPGR, RPH3A, RPS12, RRAS2, RSAD2, RSC1A1, RTN1, RTN3, RUBCN, RUSC1, RXYLT1, S100A1, S100A3, SACM1L, SAMD8, SAR1A, SAR1B, SART1, SCAMP1, SCAMP2, SCAMP3, SCAMP4, SCAMP5, SCAP, SCARA3, SCARB2, SCFD1, SCLY, SCOC, SCRG1, SCYL1, SCYL2, SCYL3, SDC1, SDC2, SDC3, SDC4, SDCBP2, SDE2, SDF4, SEC14L1, SEC16A, SEC16B, SEC22B, SEC23A, SEC23IP, SECTM1, SELENOH, SELENOI, SELENOK, SELENOM, SEMA6D, SERINC3, SERINC5, SERPINA1, SERPINB9, SFTA2, SGCE, SGMS1, SGMS2, SGSM1, SH3GL2, SH3GLB1, SH3RF1, SHH, SI, SIPA1L3, SLA2, SLC10A7, SLC16A13, SLC18A3, SLC22A13, SLC24A5, SLC26A11, SLC29A3, SLC2A1, SLC2A4, SLC30A1, SLC30A10, SLC30A5, SLC30A6, SLC30A7, SLC30A8, SLC33A1, SLC35A1, SLC35A2, SLC35A3, SLC35A4, SLC35A5, SLC35B1, SLC35B2, SLC35B3, SLC35B4, SLC35C1, SLC35C2, SLC35D1, SLC35D2, SLC35D3, SLC35E1, SLC35E2A, SLC35E2B, SLC35E3, SLC35E4, SLC35G2, SLC38A10, SLC39A11, SLC39A13, SLC39A7, SLC50A1, SLC66A2, SLC9A7, SLC9A8, SLPI, SMAD6, SMO, SMPD3, SMPD4, SNAP25, SNAP29, SNAPIN, SNTB2, SNX1, SNX17, SNX9, SOD3, SORCS1, SORL1, SORT1, SOST, SPATA16, SPEF2, SPG21, SPP1, SPPL2B, SPPL3, SPRING1, SPRR3, SPRY1, SRCAP, SREBF1, SREBF2, SRGN, ST3GAL1, ST3GAL2, ST3GAL3, ST3GAL4, ST3GAL5, ST3GAL6, ST6GAL1, ST6GAL2, ST6GALNAC1, ST6GALNAC2, ST6GALNAC3, ST6GALNAC4, ST6GALNAC5, ST6GALNAC6, ST8SIA1, ST8SIA2, ST8SIA3, ST8SIA4, ST8SIA5, ST8SIA6, STC2, STEAP2, STEAP4, STING1, STIP1, STK16, STK24, STK25, STK26, STMN2, STMN3, STMN4, STRN3, STS, STX10, STX11, STX12, STX16, STX18, STX4, STX5, STX6, STX8, SULF1, SULF2, SUN5, SURF4, SVIP, SYAP1, SYBU, SYN1, SYNDIG1L, SYNE1, SYNRG, SYS1, SYT1, SYT11, SYT17, SYT4, TAF11, TANGO2, TAPBP, TAPBPL, TAS2R16, TBC1D14, TBC1D20, TBC1D23, TBC1D5, TCP1, TDRD3, TENM1, TENM2, TENT4A, TEPSIN, TEX261, TGFB1, TGFBI, TGM4, TGOLN2, TICAM2, TJAP1, TLCD3B, TLR1, TLR2, TLR3, TLR4, TLR6, TLR7, TLR8, TLR9, TM9SF2, TM9SF4, TMBIM1, TMBIM4, TMC6, TMC8, TMCO1, TMED1, TMED10, TMED2, TMED3, TMED4, TMED5, TMED6, TMED7, TMED9, TMEM115, TMEM130, TMEM132A, TMEM165, TMEM167A, TMEM167B, TMEM192, TMEM214, TMEM230, TMEM241, TMEM30A, TMEM30B, TMEM30CP, TMEM43, TMEM50B, TMEM59, TMEM59L, TMEM79, TMEM87A, TMEM87B, TMF1, TNFRSF10A, TNFRSF1A, TNKS, TNKS2, TNRC6A, TOM1L1, TP73, TPP1, TPPP, TPST1, TPST2, TPTE2, TRAF3IP3, TRAPPC10, TRAPPC11, TRAPPC12, TRAPPC13, TRAPPC14, TRAPPC3, TRAPPC3L, TRAPPC4, TRAPPC5, TRAPPC6A, TRAPPC6B, TRAPPC8, TRAPPC9, TREML1, TRIM22, TRIM23, TRIM3, TRIM68, TRIM7, TRIP10, TRIP11, TRPC7, TRPM4, TRRAP, TSC2, TSNAX, TTC3, TVP23A, TVP23B, TVP23C, TXNDC8, UBA5, UBAC1, UBASH3A, UBIAD1, UBXN2A, UBXN2B, UGCG, UHMK1, UMOD, UNC13B, UNC45A, UNC50, UNC93B1, USO1, USP32, USP33, USP6NL, UST, UXS1, VAC14, VAMP2, VAMP3, VAMP4, VAMP5, VAMP7, VAPA, VAPB, VCAM1, VCAN, VCPIP1, VGF, VIPAS39, VPS13A, VPS33B, VPS41, VPS45, VPS51, VPS52, VPS53, VPS54, VRK1, VTI1A, VTI1B, WAPL, WDR11, WDR44, WDR77, WDR81, WHAMM, WIPI1, WLS, WNT1, WNT3, WNT3A, WNT4, WNT5A, WNT5B, WNT6, WNT7A, WNT7B, WWOX, XPR1, XYLT1, XYLT2, YES1, YIF1A, YIF1B, YIPF1, YIPF2, YIPF3, YIPF4, YIPF5, YIPF6, YIPF7, YKT6, ZDHHC1, ZDHHC11, ZDHHC11B, ZDHHC12, ZDHHC13, ZDHHC14, ZDHHC15, ZDHHC16, ZDHHC17, ZDHHC18, ZDHHC19, ZDHHC2, ZDHHC20, ZDHHC21, ZDHHC22, ZDHHC23, ZDHHC24, ZDHHC3, ZDHHC4, ZDHHC6, ZDHHC7, ZDHHC8, ZDHHC9, ZFPL1, ZFYVE1, ZG16, ZMYND8, ZNF148, ZNF622. |

\
